# Conformational, Host, and Vibrational Effects Giving Rise to Dynamic TADF Behavior in the Through-Space Charge Transfer, Triptycene Bridged Acridine-Triazine Donor Acceptor TADF Molecule **TpAT-tFFO**

**DOI:** 10.1021/acs.jpcc.2c07529

**Published:** 2023-04-27

**Authors:** Hector Miranda-Salinas, Angela Rodriguez-Serrano, Jeremy M. Kaminski, Fabian Dinkelbach, Nakagawa Hiromichi, Yu Kusakabe, Hironori Kaji, Christel M. Marian, Andrew P. Monkman

**Affiliations:** † OEM Research Group, Department of Physics, Durham University, Durham DH1 3LE, UK; ‡ Institut für Theoretische Chemie und Computerchemie, Heinrich-Heine-Universität Düsseldorf, Universitätsstraße 1, D-40225 Düsseldorf, Germany; § Institute for Chemical Research Kyoto University, Uji, Kyoto 611-0011, Japan

## Abstract

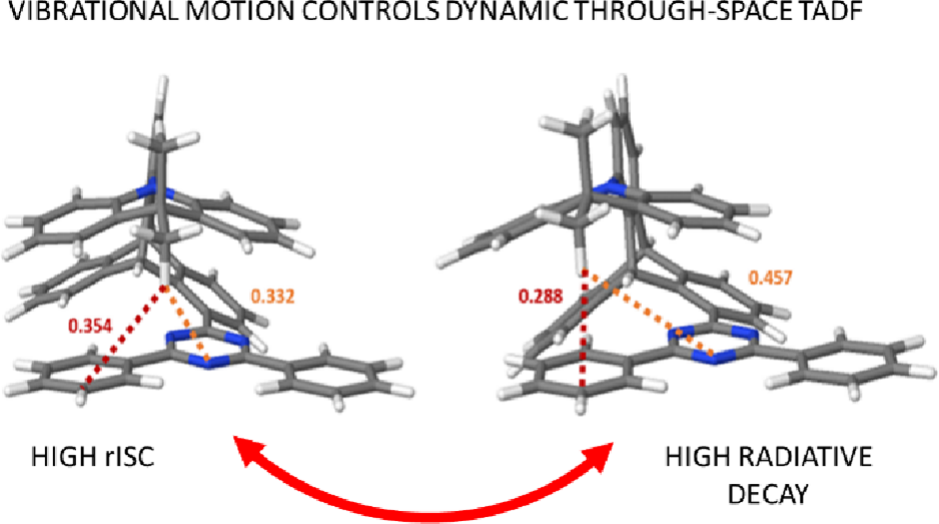

We present a joint experimental and theoretical study of the through-space charge transfer (CT) TADF molecule **TpAT-tFFO**. The measured fluorescence has a singular Gaussian line shape but two decay components, coming from two distinct molecular CT conformers, energetically only 20 meV apart. We determined the intersystem crossing rate (1 × 10^7^ s^–1^) to be 1 order of magnitude faster than radiative decay, and prompt emission (PF) is therefore quenched within 30 ns, leaving delayed fluorescence (DF) observable from 30 ns onward as the measured reverse intersystem crossing (rISC) rate is >1 × 10^6^ s^–1^, yielding a DF/PF ratio >98%. Time-resolved emission spectra measured between 30 ns and 900 ms in films show no change in the spectral band shape, but between 50 and 400 ms, we observe a *ca.* 65 meV red shift of the emission, ascribed to the DF to phosphorescence transition, with the phosphorescence (lifetime >1 s) emanating from the lowest ^3^CT state. A host-independent thermal activation energy of 16 meV is found, indicating that small-amplitude vibrational motions (∼140 cm^–1^) of the donor with respect to the acceptor dominate rISC. **TpAT-tFFO** photophysics is dynamic, and these vibrational motions drive the molecule between maximal rISC rate and high radiative decay configurations so that the molecule can be thought to be “self-optimizing” for the best TADF performance.

## Introduction

A new generation of triplet harvesting organic light-emitting diodes (OLEDs) uses thermally activated delayed fluorescence (TADF) emitter molecules to achieve nearly 100% internal efficiency,^1^ without using heavy metals, via the mechanism of reverse intersystem crossing (rISC), harvesting the lowest energy triplet state (^3^CT) to the singlet state (^1^CT).^2^ However, when the energy difference between these states approaches zero, the ^1^CT ↔ ^3^CT interconversion becomes spin-forbidden because the orbital angular momentum cannot change during the transition.^3^ Recently, we have shown that in organic TADF molecules, even when the ^3^CT and ^1^CT energy gap (Δ*E*_ST_) is small (<50 meV), rISC can be very efficient due to rISC mediated by a third triplet excited state through a non-adiabatic vibronic coupled spin–orbit coupling mechanism.^4,5^ Efficient TADF molecules usually have charge transfer excited states with effective spatial decoupling between the electron in the lowest unoccupied molecular orbital (LUMO) and its partner in the highest occupied molecular orbital (HOMO), which minimizes the electron exchange energy and Δ*E*_ST_.^6^ This is most commonly achieved by conformational twisting of the donor (D) relative to the acceptor (A) moieties, typically about a N–C bridging bond that naturally introduces a large dihedral angle, approaching 90°, giving effective decoupling of the electron and hole.^7^ This is an example of a through-bond charge transfer (TBCT) across a physical (conjugating) bridge bond between D and A.^8,9^ An electronically decoupled D–A charge transfer state can also form between separate D and A molecules when they are in close contact, forming a bimolecular exciplex state. In some cases, these exciplexes also show efficient TADF through the same non-adiabatic vibronic coupled spin–orbit coupling mechanism.^10^ In this case, the electronic decoupling is achieved by a large physical spatial separation of the electron and hole in the excited state. This is an example of through-space charge transfer (TSCT), CT between the physically separated D and A. In both cases, the excited CT state has a very high dipole moment and is highly sensitive to its external environment, such as solvent polarity, leading to large solvatochromic shifts in solutions of increasing solvent polarity.^11,12^

Alternatively, TSCT spatial separation can be achieved with an inert scaffold unit acting as the bridge between D and A units. Here, the spatial separation must be small enough to maintain some π-wavefunction interaction between them.^13,14^ In recent years, studies on TSCT have led researchers to come forward with designs that help to optimize this kind of CT state^9,15,16^ based on the success of exciplex systems that yield very efficient TADF but in uncontrolled and highly inhomogeneous systems.^17^ The strategy used by Wada *et al*(18) in **TpAT-tFFO** is by far better in this regard using D and A moieties that yield efficient second-order vibronic coupled spin–orbit coupling,^4,19,20^ combined with an (electronically inert) triptycene scaffold to optimize the spatial separation in a tilted face-to-face (tFF) alignment of acceptor and donor moieties, with an optimized separation distance (tFFO), a key factor to develop new and more efficient TSCT TADF materials. The system fulfills the requirements for a non-adiabatic vibronic coupled spin–orbit coupling mechanism, i.e., having near degenerate ^1^CT, ^3^CT, and ^3^LE excited states. Recent studies on other TSCT systems have shown that the competition between TBCT and TSCT in a molecule can occur and also that the scaffold bridge unit can be involved in the CT states.^21^

**Figure 1:**
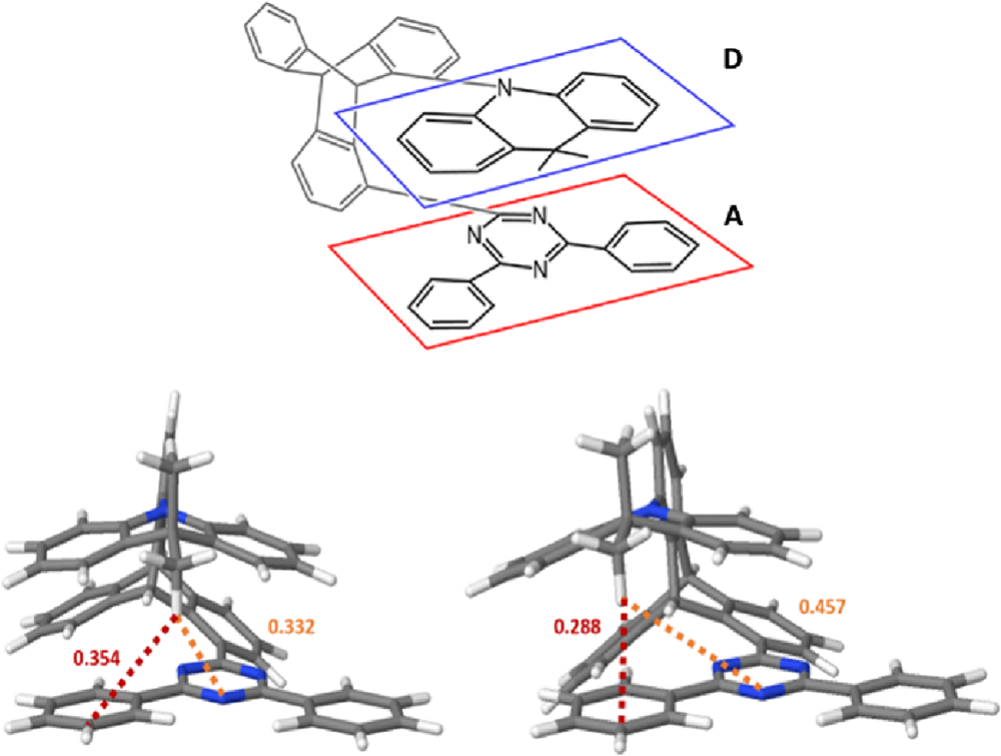
Chemical structure of **TpAT-tFFO** and the two lowest energy conformers found computationally, named S_0_ (Me → N) and S_0_ (Me → Ph). The conformers were named after the distances between the proximal methyl hydrogen atom of the **DMAC** donor and the triazine (orange) or phenyl (red) rings of the acceptor, given in nanometers.

Here, we present in-depth photophysical studies combined with results from high level DFT-MRCI theoretical calculations of **TpAT-tFFO**, which are described in detail in our sister paper,^22^ exploring the behavior of the TSCT states in different solvents and solid host matrix environments to fully understand TADF from such controlled TSCT molecules. The **TpAT-tFFO** molecule is composed of 9,9-dimethyl-9,10-dihydroacridine (**DMAC**) and 2,4-diphenyl-1,3,5-triazine (**dPT**) as D and A, respectively (Figure 1). These units are known to give CT states in a variety of D–A molecules^23^ and D–A exciplexes. As an inert scaffold (bridge), a triptycene (**Tp**) unit is used to obtain the desired optimal spatial separation of D and A units.^24,25^ Using triptycene as a scaffold allows the D and A to take up a tFF configuration, which is believed to be critical for efficient magnetic coupling required for high spin–orbit coupling (SOC) and efficient TADF.^18^ Our computational analysis has identified two distinct stable low energy conformers of **TpAT-tFFO** (Figure 1), which however complicates the photophysics of **TpAT-tFFO**. These have very similar electronic structures and energies but have a large energy barrier for interconversion in the excited state, which should manifest in the solid-state photophysics of the material.

## Methods

### Steady State

Photoluminescence measurements were obtained using drop cast films on sapphire substrates at 1% by weight for zeonex and 10% by weight for the other hosts, and for the solution measurement, concentrations of 20 μML^–1^ were used. A Jobin Yvon Horiba Fluoromax-3 and a spectrophotometer Shimadzu UV–vis–NIR 3600 were used for emission and absorption measurements, respectively. All spectral onset energies were corrected using the Jacobian conversion of wavelengths to energies.

### Time-Resolved Measurements

The time-resolved measurements were obtained using a gated iCCD camera (250-950 nm) system, and for the temperature-dependent measurements, a helium-closed cycle cryopump, with optical windows, Si thermodiode, and sample mount, attached directly to the cold head. TCSPC measurements were recorded with a Horiba DeltaFlex TCSPC system using a Horiba NanoLED 357 nm and SpectraLED 330 nm as light sources.

### Computations

The computational protocol closely resembles the one followed in ref (26). In short, (time-dependent) density functional theory PBE0/SV(P) was utilized to compute equilibrium geometries and vibrational frequencies whereas excitation energies, transition dipole moments, and wavefunctions for subsequent spin–orbit coupling calculations were computed using a multireference configuration interaction approach employing the DFT/MRCI-R2016 Hamiltonian. Rate constants of radiative and nonradiative transitions were calculated including vibronic interactions at the Herzberg–Teller level of theory. For programs and further technical details of the calculations, we refer to a sister paper.^22^

## Results

**Figure 2:**
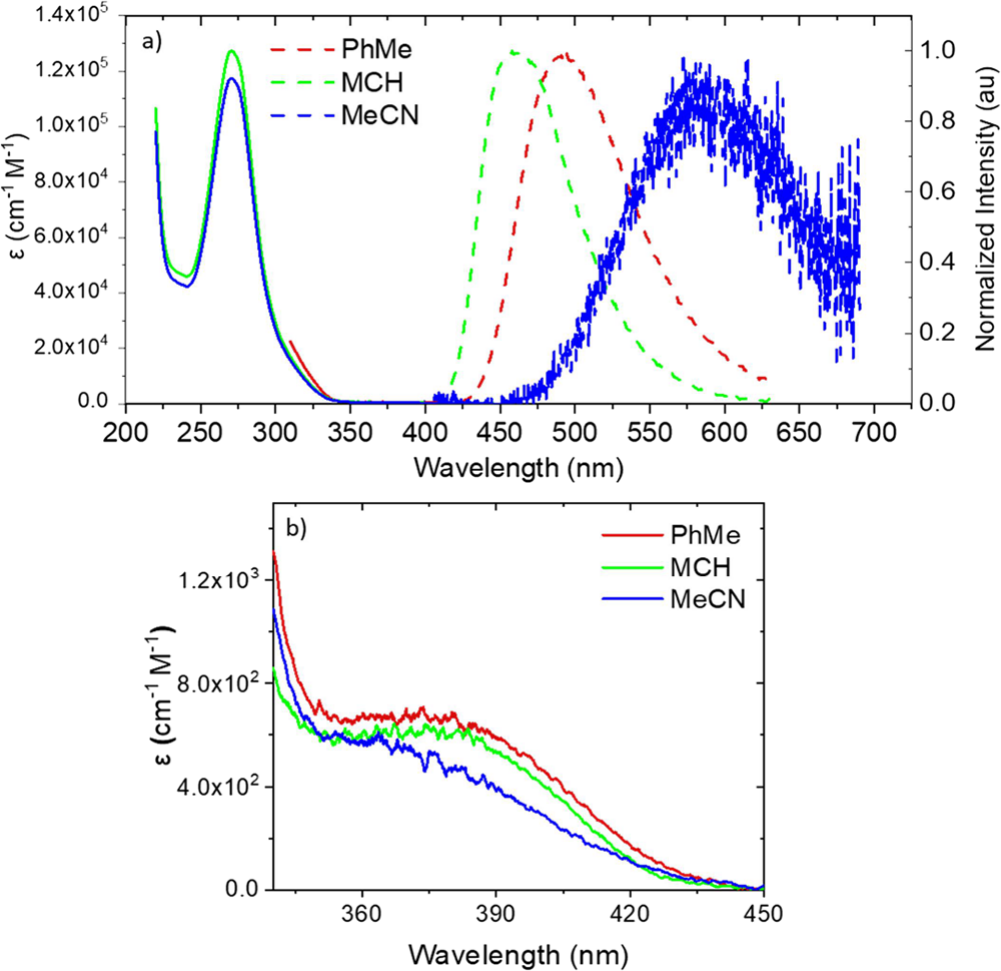
(a) Solution-state PL measurements, solid lines correspond to absorption and dashed lines correspond to emission (toluene absorption spectra were only 310 nm because of the solvent cutoff). (b) Absorption in the spectral region of 340–440 nm showing a weak solvent-dependent direct charge transfer transition below the lowest energy ππ* transition.

Absorption and emission spectra of **TpAT-tFFO** were measured in three different solvents (aerated and degassed): methylcyclohexane (MCH), toluene (PhMe), and acetonitrile (MeCN) (Figure 2a,b). The main absorption peak is seen at 271 nm (ε = 1.3 × 10^5^ cm^–1^ M^–1^), which matches well with both the absorption band of the **DMAC** donor unit^27^ and the triphenyltriazine acceptor band.^14^ Calculations confirm that the local ππ* excitations of both the acceptor (285 nm) and donor (275 nm) occur in this spectral region.

**Figure 3:**
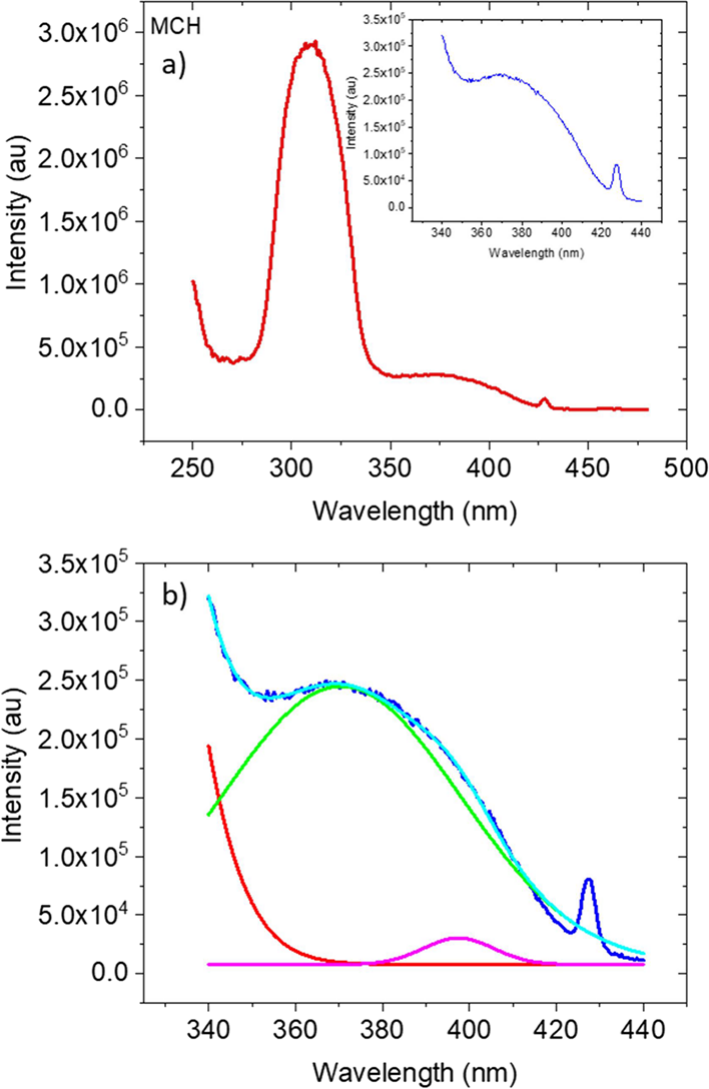
(a) Excitation spectrum of **TpAT-tFFO** dissolved in MCH; the inset shows well-resolved direct CT absorption below 340 nm and (b) close inspection of the 340–420 nm band (dark blue) fitted (light blue) with two exponential components (green and pink). The red curve represents the tail of the S_3_ band. The spike at 427 nm is a solvent Raman band with a monitored emission wavelength of 490 nm.

We further observe an absorption feature on the red edge of these strong ππ* bands, at *ca.* 300–320 nm (ε = 7.5 × 10^3^ cm^–1^ M^–1^), which is more clearly seen in excitation spectra monitored at the peak of the CT emission band (Figure 3a and Figures S1–S6). Computational studies reveal this transition (labeled S_0_–S_3_) to be a composite transition from the triptycene bridge to the triazine CT state mixed with a local excitation on the triptycene, having moderate oscillator strength ε = 5 × 10^3^ cm^–1^ M^–1^. Excitation spectra in aerated solution (Figure S7a) show that this transition gives prompt CT emission by pseudo-TBCT excitation. The excitation-dependent CT emission is constant for excitation between 300 and 370 nm (Figure S7b), showing that the triptycene bridge is involved in low energy excitations of the molecule. Further, excitation of the 300–320 nm band gives a weak but measurable emission in the range of 350–425 nm (Figure S7b), whereas excitation above 320 nm gives no such emission. This we believe arises from the triazine ππ* state before charge separation occurs,^21^ indicative of the weak through-space electronic coupling between triazine and acridine units, allowing the triazine decay to compete to a small degree with the electron transfer step. Figure 2b and Figures S4–S6 also show a further, very low extinction absorption feature, *ca.* ε = 7 × 10^2^ cm^–1^ M^–1^ below this transition in the range of 340–420 nm, which we associated with direct through-space CT absorption. Again, this band is more clearly seen in the excitation spectra (Figures S1–S3). Gaussian deconvolution of the absorption and excitation spectra (Figure 3b and Figures S1–S3) reveals two Gaussian components, peak wavelengths of 370 and 395 nm, with extinction coefficients of *ca.* 6 × 10^2^ and 3 × 10^2^ cm^–1^ M^–1^, respectively. The main 370 nm feature is assigned to the lowest lying S_0_–S_1_ and S_0_–S_2_ CT transitions of each conformer. Calculations suggest that depending on the molecular conformer, the ratios of extinction coefficients between the 275 nm ππ* transition and these transitions will be *ca.* 10 for the S_0_ (Me → N) conformer and *ca.* 40 for the S_0_ (Me → Ph) conformer. Experimentally, we find a ratio very close to 40 between the two transitions (Figure 2b), indicating a predominance of the slightly more stable but higher CT energy S_0_ (Me → Ph) conformer at room temperature in solution. However, we cannot spectrally resolve the S_0_–S_1_ and S_0_–S_2_ pair of (direct CT) absorptions in solution (Figure 3b). Calculations suggest that the difference of the S_0_–S_1_ and S_0_–S_2_ energies for the conformers is only 20 meV. The extremely weak band observed at *ca.* 400 nm may be due to residual dimers/aggregates. However, excitation at this wavelength still results in an emission spectrum identical to **TpAT-tFFO** CT emission (Figure S4), most likely due to the residual tail absorption from the direct CT transitions.

In highly polar MeCN, we only observe a 360 nm band. These direct CT absorption bands show a little (instantaneous) blue shift with increasing solvent polarity, potentially indicating nπ* character. Degassed excitation spectra (250–425 nm) show a uniform increase by nearly a factor of 100, indicating the very high DF contribution to the total emission spectra (Figure S4a).

The onsets of CT emission in MCH, PhMe, and MeCN are found to be 2.99, 2.86, and 2.57 eV, respectively (Figure 2a). Emission in MeCN is very weak compared to that observed in the less polar solvents. The solution measurements were made both in the presence of oxygen and degassed, and the effect of degassing the solutions is shown in Figure S5 where it is seen that oxygen quenches the CT emission very effectively, indicating the very large contribution of delayed CT emission to the overall luminescence of **TpAT-tFFO**. The largest DF contribution is observed in PhMe, accounting for some 98.70% of the total luminescence, followed by MCH with 93.62% and the smallest in MeCN with 88.87% (Table 1) and indicates very fast intersystem crossing rates from ^1^CT, much faster than radiative decay rates.

**Table 1:** Degassed and Oxygenated Solution Measurement Data

solvent	τ_PF1_ [ns]	τ_PF2_ [ns]	τ_DF1_ [μs]	τ_DF2_ [μs]	*k*_F_ [10^5^ s^–1^]	*k*_ISC_ [10^6^ s^–1^]	*k*_rISC_ [10^5^ s^–1^]	DF/PF
MCH	8.0 ± 0.42	70.88 ± 18.89	1.64 ± 0.03		19.05 ± 1.48	11.31 ± 0.28	11.29 ± 1.00	93.62%
PhMe	7.9 ± 0.77	48.9 ± 6.37	4.4 ± 0.10		34.70 ± 1.91	12.54 ± 2.75	31.91 ± 1.63	98.7%
MeCN	14.71 ± 1.45	88.26 ± 26.60	0.62 ± 0.01	16.2 ± 0.65	36.89 ± 0.00	9.81 ± 0.00	0.74 ± 0.00	88.87%

To observe the differences that exist in the molecule while changing the medium, steady-state emission measurements were done in films for **TpAT-tFFO**; same as in solution, the compound shows only CT emission, with onsets at 2.96, 2.89, 2.86, and 2.93 eV for zeonex, UGH-3, mCBP, and CzSi host matrices, respectively. The carbazole containing hosts (mCBP and CzSi) show an additional emission peak at 371 nm, coming from the host because of the unavoidable overlap of host and guest absorption bands (Figure S6). The steady-state emission in the hosts shows little correlation between host properties such as dielectric strength and the CT energy in this molecule.^28^

**Figure 4:**
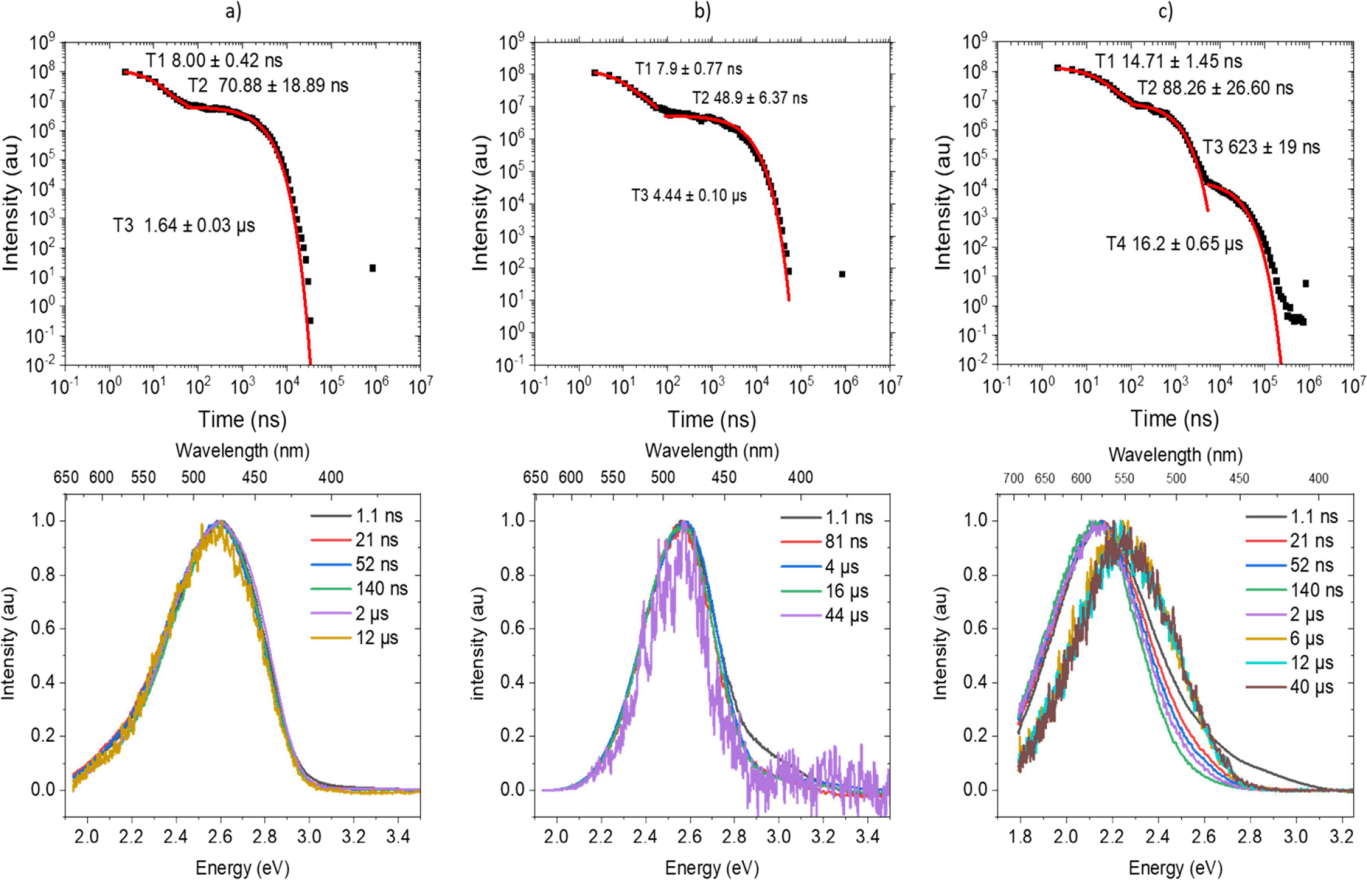
Time-resolved degassed measurements of **TpAT-tFFO** in (a) MCH, (b) PhMe, and (c) MeCN solutions.

**Figure 5:**
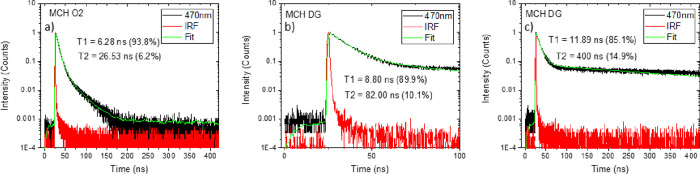
TCSPC decay measurements of **TpAT tFFO** in MCH measured in (a) aerated solution, (b) degassed, and (c) degassed in a longer time range.

Time-resolved **TpAT-tFFO** emission in solution shows classic TADF decay kinetics. In toluene and MCH, we observe initial fast prompt and then delayed CT emission. However, the prompt decay has two decay components with lifetimes of 8 and 50–70 ns but only one Gaussian emission band. The delayed CT emission is mono exponential with a solvent-dependent lifetime of 1.6–4.4 μs (Figure 4a,b and Figures S7 and S8). In aerated solutions, the DF emission is effectively quenched and only the two-prompt decay components are observed. Time-correlated single-photon counting (TCSPC) measurements in MCH (Figure 5a,b, toluene; Figure S7) are rather enlightening. In aerated MCH solution, we confirm two prompt decay lifetimes of 6.3 ns (94%) and 26.5 ns (6%); toluene is almost identical. In degassed MCH, the DF decay is very strong being the dominant component from about 30 ns onward, see the plateau in the TCSPC decays in Figure 5b,c. This completely skews the estimation of the second “prompt” decay time in degassed conditions, especially given its very low amplitude. However, in degassed solution, we observe the same emission spectra from 1 to 12–44 μs (Figure 4b). Given that we observe that DF dominates after *ca.* 30 ns, this implies that radiative decay of the prompt ^1^CT state is very slow and so rapidly quenched by much faster ISC; in this kind of long-lived prompt emission, the presence of self-quenching by oxygen has been reported before.^29,30^ Also, rISC must be fast as well. Clearly, this TSCT **TpAT-tFFO** molecule has strong magnetic coupling between its CT and locally excited (LE) states. The relatively fast rISC then produces enough DF emission to be observed after 30 ns, but the rISC process still has a lifetime in the microsecond regime; simply put, it is not ultrafast. As all the prompt signals are quenched, we can see the DF signal from very earlier times.

From our calculations, we ascribed the two ^1^CT decay components at the same emission wavelengths to arise from the two conformers of **TpAT-tFFO** (Figure 1). In the ground state, the energy barrier between the two conformers is shallow, allowing rapid interconversion between the two, especially in solution. However, in the excited state, the energy barrier for interconversion is much larger because of the larger molecular structural rearrangement required, allowing us to observe both emission decay components, but for long time DF emission, only one conformer contributes, as will be described later.

Taking the fluorescence quantum yield measured by Wada *et al.*(18) in toluene, 84%, and the prompt lifetimes of the two conformers in aerated toluene measured by TCSPC, 8.4 and 30 ns, we calculate from the ratio of the areas of steady-state emission measured in aerated and degassed solutions that the PLQY of the prompt emission component is 2% (Figure S5). From this, we obtain radiative lifetimes of 420 ns and 1.5 μs and radiative decay rates of 2.38 × 10^6^ and 6.67 × 10^5^ s^–1^, respectively, for the two conformer species. These are in good agreement with the rates determined from our kinetic fitting (Table 1) and with our calculated S_1_ → S_0_ (Me → N) radiative decay rate of 7.0 ×10^5^ s^–1^ and S_2_ → S_0_ (Me → Ph) radiative decay rate of 2.0 × 10^5^ s^–1^. These are very long indicative of weak coupling between the CT and ground state.

In MeCN, we observe more complicated photophysics with lifetimes and spectral components highly modified by the strongly polar environment (Figure 4c and Figure S9a) and are discussed in the Supporting Information.

Kinetic model fitting^29^ of the decay curves yields rISC rates for the DF observed, in toluene of 3.2 × 10^6^ and 1.1 × 10^6^ s^–1^ in MCH, which are very fast. In MeCN, taking the slow decay component to be true DF, we calculate a rISC rate of *ca.* 7.5 × 10^4^ s^–1^, in line with a much larger singlet–triplet gap. Data for **TpAT-tFFO** in degassed solution is given in Table 1. The peak in the DF/PF ratio in toluene indicates that in low polarity media, the ^3^CT state lies above the (mediating) ^3^LE state. In toluene, the energy gap between them is at its smallest, while in MeCN, the ^3^CT has dropped below ^3^LE opening up the singlet–triplet energy gap again, a behavior seen in many TADF materials.^4^

**Figure 6:**
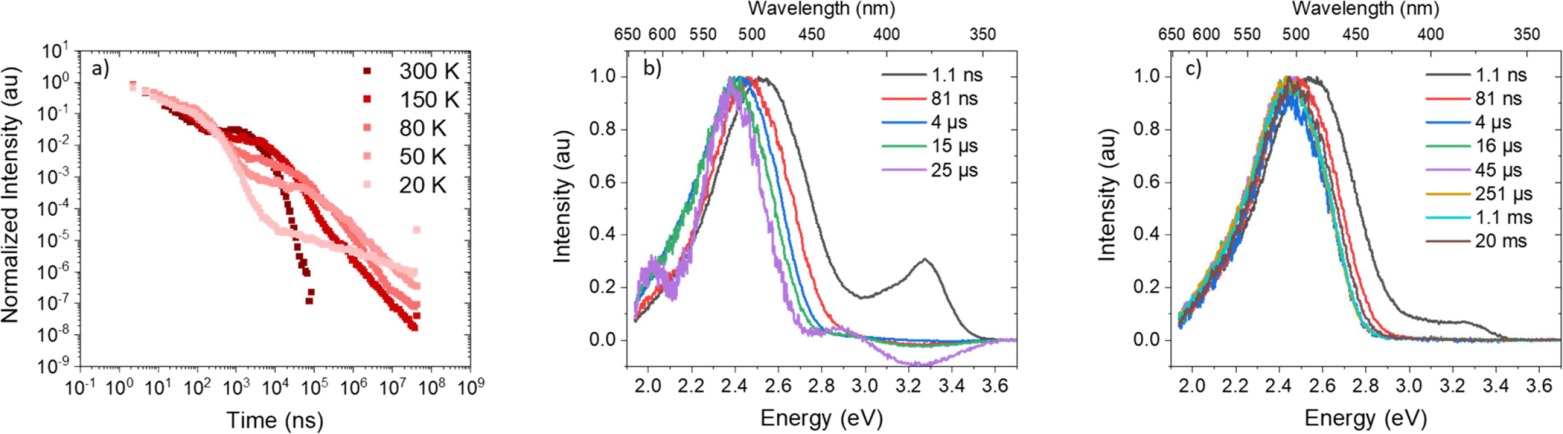
(a) Temperature-dependent decays and spectra of **TpAT-tFFO** using a mCBP matrix. Emission observed at very early times in the 350–425 nm region comes from the host mCBP as the absorption of the host overlaps with the **TpAT-tFFO** at the 355 nm laser excitation wavelength, at temperatures of (b) 300 K and (c) 50 K. Further temperatures are shown in Figures S14 and S15.

**Figure 7:**
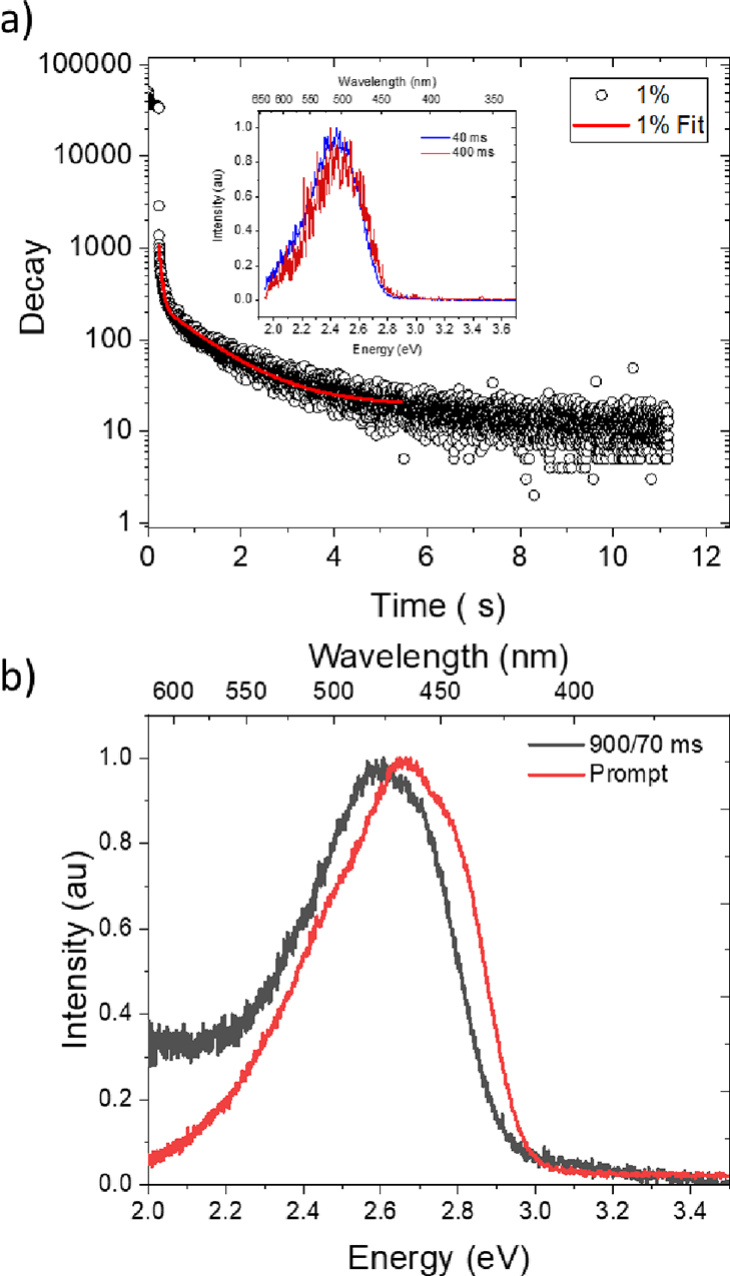
(a) Long-time decay of emission from a mCBP 1% **TpAT-tFFO** film fitted with a biexponential curve with lifetimes of 56 ms and 1.1 s. The inset shows the emission spectra recorded at 40 and 400 ms, showing that the two decay components come from energetically identical species. (b) Comparison of prompt fluorescence with 900 ms delayed phosphorescence measured at 20 K for a **TpAT-tFFO** zeonex film.

Time-resolved measurements of **TpAT-tFFO** in various hosts were also made as a function of temperature. All rate constants and lifetimes were determined using the fitting method reported by Haase *et al*.^31^ Measurements were made from 300 to 20 K. For **TpAT-TFFO** (10%) in the mCBP host (Figure 6b shows the 300 K decay and spectra), the emission has an onset energy of 2.818 ± 0.005 eV, which rapidly red-shifts over the first 100 ns by *ca.* 150 meV. This shift is found to be both host- and temperature-dependent but is not observed in MCH or toluene solution measurements. At 300 K, both prompt and delayed fluorescence CT decays are faster than at lower temperatures, showing that both ISC and rISC rates are thermally activated processes (Table 2). We also observe that the red shift of the CT state is less pronounced at low temperature (Figure 6b,c), which we take as an indication that in solid-state hosts, the D and A units have some conformational inhomogeneity yielding a distribution of CT energies and rISC rates.^26^ At 300 K, a rISC rate of 1.1 × 10^6^ s^–1^ is found in mCBP (Table 2). As the temperature is reduced, both ISC and rISC rates decrease, showing that ISC is also a thermally activated process, governed by a second-order vibrational coupling mechanism controlling ^1^CT to ^3^CT ISC in the direct analogue to rISC, the first clear observation of this fact in TADF materials.^3^ We also observe the onset of a long-time non-exponential tail in the DF emission at low temperature, even at 20 K. A very similar behavior is observed in the other small molecule hosts, CzSi and UGH-3 (Table 3 and Figures S11–S13, S16, and S17 and Tables S1–S3). In mCBP at 20 K strong phosphorescence emission, an onset at 2.9 ± 0.005 eV is observed (even at 900 ms time delays; Figure 7c).

**Table 2:** Rate Constants for the Different Temperatures of the mCBP Matrix Time-Resolved Measurements

mCBP (10%)			
*T* [K]	*K*_F_ [10^5^ s^–1^]	*K*_ISC_ [10^6^ s^–1^]	*K*_rISC_ [10^5^ s^–1^]
300	8.22 ± 0.1	8.97 ± 0.11	11.21 ± -
150	4.99 ± 0.06	7.29 ± 0.10	3.56 ± -
80	3.97 ± 0.03	2.05 ± 0.15	0.48 ± 0.04
50	6.98 ± 0.55	1.00 ± 0.02	0.11 ± -

**Table 3:** Onset Energies Measured Using Different Hosts: Zeonex, UGH-3, mCBP, and CzSi

host	^1^LE (eV)	^1^CT (eV)	^3^CT (eV)	Δ*E*_ST_ (eV)
UGH-3		2.853	2.839	0.014
mCBP	3.495	2.818	2.800	0.018
CzSi	3.496	2.893	2.877	0.016
zeonex	3.489	2.943	2.930	0.013

In all solid-state hosts, room temperature rISC rates are above 1 × 10 ^6^ s^–1^ but not as high as found in toluene. We find that the energies of the ^1^CT state change slightly from host to host, but from the temperature-dependent ISC and rISC rates, we determine an identical thermal activation energy for the non-adiabatic coupling^19,32^ of *ca.* 17 meV (Figure S22), independent of the host. The vibrational mode that couples the triplet states to mediate the rISC mechanism is calculated to be the 1600 cm^–1^ (200 meV) breathing mode of the triazine unit, whereas 17 meV corresponds to a vibrational mode of 140 cm^–1^. Our calculations indicate that such low energy torsional modes of D with respect to A units greatly affect both the Δ*E*_ST_ gap and SOCME, and so, freezing-out of these vibrational motions at low temperatures has a large effect on the rISC rate. This would explain the host-independent behavior of this activated process. As the temperature is reduced, we see a smaller monotonic red shift over the DF lifetime, and below 150 K, the DF emission is always *ca*. 50 meV higher than observed at 300 K. This is in line with the interconversion of the two conformers that requires thermal energy to overcome the large energy barrier of the structural reorganization. Thus, less of the lower GS energy conformer (Me → Ph), which has a higher CT energy, will interconvert to the high GS energy but lower CT energy (Me → N) species at low temperatures in the solid state, giving rise to the bluer DF emission at low temperatures. The prompt relaxation over 400 ns is then representative of contributions from the fast and slow conformers with much slower interconversion than in solution (Figures S12 and S13), along with the effects of inhomogeneity causing a distribution of rISC rates in both cases.

Importantly, in zeonex films, we observe only a very small red shift (20–30 meV) of the CT state energy from 1 ns (our time resolution) to 20 ms (Figures S19 and S20), in line with a very homogeneous D–A spatial conformation. As in MCH solution, the prompt emission has two decay components, *ca.* 8.1 and 100 ns, along with a mono exponentially decaying DF with a lifetime of 5.8 μs. Given that zeonex is a low-density amorphous polymer having non-symmetrical branched side chains, it therefore has a great deal of free volume to allow the D and A units of the **TpAT-tFFO** molecule to rearrange very rapidly after photoexcitation compared to the more hindered motion in the tightly packed small molecule host matrices. In this sense, zeonex acts very much like a viscous fluid and so the **TpAT-tFFO** behaves as in solution. In the time-resolved heat maps measured in zeonex, we observe a very small red shift (less than 5 nm), which could indicate the two conformer populations reaching equilibrium, but on a slower time scale than in MCH in line with the much greater “viscosity” of zeonex. Also, as with the small molecule hosts, we do observe the grow-in of a power law, long life-time DF tail (Figure S11). In all solid-state hosts, the rISC rate seems rather independent of the host.

Finally, using **TpAT-tFFO** zeonex and mCBP films at 20 and 80 K, respectively, time-resolved spectra at very long decay times were measured (Figure 7a and Figures S15 and S21). We observe no change in emission spectra from 30 ns until 50 ms, DF having the same onset energy as ^1^CT prompt emission. In zeonex at 20 K, a gradual red shift from 50 ms until 400 ms is observed. Emission can be seen by eye even after 10 s, and our lowest laser repetition rate is 1 Hz, so we are limited to 900 ms delay time measurements. This ultralong-lived emission has an invariant spectral band shape. No change in the vibronic structure is seen, even at 900 ms. Comparing the band onset at 50 ms to that at 900 ms, we estimated a red shift of 50–75 meV (Figure 7b). Fitting the long-time emission decay in mCBP measured at 80 K gives two lifetimes, 50 ms and 1.1 s. This we believe shows the emergence of phosphorescence from the DF at 50–100 ms.

## Discussion

In the solution-state measurements, we observe direct CT absorption indicating a ground state through-space interaction via the π-wavefunction overlap between D and A units in **TpAT-tFFO**. These bands show weak negative solvatochromism, indicating some n−π mixing in the orbitals involved. Clear TADF is observed from these CT states, and toluene gives an extremely large DF/PF ratio (98.7%), indicating the dominance of ISC over the radiative decay of the singlet CT state. Taking the lowest triplet energy from solid-state phosphorescence measurements, we estimated a very small singlet–triplet energy gap (<20 meV) in toluene, optimal for the largest rISC rate we observe. Such a small S–T gap indicates that the local ^3^LE triplet state must be mediating the rISC in this TSCT system. **TpAT-tFFO** emission in toluene and MCH shows no spectral shift over all measurement times, yet the PF is biexponential. This is explained by the presence of two structural conformers, having nearly degenerate CT energies but different (slow) radiative decay rates, as we find from our computational modeling, where an energy difference of only 20 meV between energy states of each conformer is calculated. In both species, the ISC is an order of magnitude faster than radiative decay and so rapidly quenches the slow prompt emission. From TCSPC, we see the effect of this rapid quenching in that DF is observed to take over emission from 30 ns *k*_rISC_ = 3.2 × 10^6^ s^–1^, but as the prompt emission is quenched within 30 ns, we observe DF from this time onward. The calculated value for *k*_ISC_ = 1 × 10^7^ s^–1^ is in good agreement with the experimental value obtained here. In **TpAT-tFFO**, prompt emission is rapidly quenched by fast ISC, but rISC is also fast and rISC is very effective. Thus, the DF contribution is seen to be the major contribution to emission, see Figure S8. Hence, in toluene, where the exchange energy is minimal, the prompt component is very small but DF is very high, so overall, the PLQY is very high. PLQY is also enhanced because the ^3^CT state is the lowest triplet state of the molecule. All triplets are effectively trapped in this state having both very weak very long-lived phosphorescence, which also indicates that non-radiative decay is virtually zero. The ^3^CT state therefore acts as a triplet reservoir, allowing almost all triplets to be up-converted to the singlet state by the rISC mechanism.

Both conformers are found to have very long radiative lifetimes, 420 ns for the S_2_ (Me → N) conformer and 1.5 μs for the S_1_ (Me → Ph). These states have a large energy barrier for interconversion in the excited state; however, in the ground state, calculations show that vibrational torsional motion of the D with respect to the A units effectively drives interconversion of the conformers.

In the solid state, unsurprisingly, zeonex gives a very similar CT energy to MCH solution, with <5 nm red shift over time. Meanwhile, small molecule hosts that pack more closely hinder possible molecular reconfiguration and motion and we observe a slow red shift over tens of nanoseconds of the prompt emission. At low temperature, this relaxation slows down further, indicating possible interconversion of conformers or simple energy relaxation through a small thermally activated D–A rearrangement required to overcome the large reorganization energy between the two forms. As the (Me → Ph) conformer is slightly more stable in the electronic ground state, we should expect an increase in population (of this higher energy CT state) at lower temperatures relative to the (Me → N) conformer. These long-lived (Me → Ph) conformers give rise to the increasing (blue-shifted) long-time DF tail and bluer overall DF emission that we observe at low temperature, indicative of less efficient slower rISC from these species.

In the CzSi host, we observe only a very small time-dependent red shift at all temperatures, confirming the nature of the host molecule packing and its interactions with the emitter controlling this mechanism. High rISC rates are found in all solid-state hosts, above 1 × 10^6^ s^–1^ and reaching 2.6 × 10^6^ s^–1^ in UGH-3. In all cases, we observe that both ISC and rISC are temperature-dependent, clearly showing that both are mediated by vibronic coupling. From RT to 20 K, the rate of ISC decreases by an order of magnitude, whereas the rISC rate decreases by around 2 orders of magnitude, which indicates different mechanisms controlling these processes.

Given the similar small energy gaps found in all hosts, especially at room temperature, UGH-3 stands out with a rISC rate greater by a factor of 2 compared to the other hosts. As all hosts used have low polarizability, we believe that UGH-3 packs with **TpAT-tFFO** to give a more optimal D–A spatial overlap configuration that enhances the magnetic coupling (SOC) between them. This might not be the lowest energy equilibrium geometry of the molecule however but indicates the way to optimize TSCT rISC, e.g., by stabilizing the (Me → N) fast rISC conformer, for example, in the case of **TpAT-tFFO**.

Our calculations reveal that the (Me → Ph) conformer has lower SOC and vibronic coupling at lower temperature, and this supports our idea that a growing population of (Me → Ph) conformers at low temperature gives rise to the observed increasing long-time non-exponential tail DF contribution. The conformers are unable to interconvert because of the host packing and lack of thermal energy to drive the interconversion over the large energy barrier in the excited state. Calculations also show that the optical transition probability is very sensitive to the singlet–triplet energy gap, and at low temperature, any frozen-in inhomogeneity will cause a large dispersion in the rISC rates. Thus, at room temperature, we observe no long-lived DF tail because all slow rISC (Me → Ph) conformers can convert to fast rISC ones (Me → N). Also, in solution, this conversion is fast so no tail is observed as well. In this case, thermal disorder is minimized at RT and the long-lived DF tail is greatest at low temperature. The vibrational mode that drives the interchange between the two conformers is shown in Figure S24 and can best be described as a torsional rocking motion of the D with respect to the A. From the experimental verification of the two interconverting near isoenergetic conformers having very different radiative lifetimes, ISC and rISC rates combined with the theoretical identification of the sensitivity of these parameters to the separation and orientation of the D and A units, we see that vibrational motion must play a very important role in dictating these important rates.

Further, at high temperatures, this thermal motion will enable each molecule to dynamically access a range of D and A spatial configurations such that both a fast rISC rate and a fast radiative decay rate molecular configuration can be dynamically accessed on the vibrational time scale. Thus, both highly efficient emission (PLQY >90%) and fast rISC (>2 × 10^6^ s^–1^) can be achieved simultaneously. Calculations show that displacements along the low frequency mode 1 (Figure S27) accomplish the interconversion of the conformers in the electronic ground state and that two further low frequency vibrational modes, 9 and 12 (Figure S25), mostly affect the donor–acceptor interplanar distance and thus the Δ*E*_ST_ gap, oscillator strength, and rISC rate. These latter modes effectively cause rocking and distortion of the D and A units such that a D–A pair moves closer and further apart from each other and changes face-to-face orientation dynamically, changing the D–A wavefunction overlap. Our calculations indicate that vibrational motion along any of these three modes readily leads to S_1_–T_3_ crossing, i.e., ^1^CT-^3^LE mixing (Figures S24 and S25), with mode 1 also causing large changes in the SOCME for the S_1_–T_3_ transition. Further, mode 9 and mode 12 have a major impact on the oscillator strength of the S_1_ and S_2_ states, respectively. Experimentally, we observe a host-independent thermal activation energy of 17 meV (140 cm^–1^) fully in-line with reduced rISC because these low energy torsional modes are frozen out. These observations and calculations together strongly suggest that these thermally driven vibrational motions of the molecule (relative D–A motions) enhance rISC rates, not only by increasing vibronic coupling but also by allowing the molecule to sample very small ^1^CT-^3^LE and ^3^CT-^3^LE Δ*E*_ST_ gap configurations. This motion also enhances rISC by increasing the SOC coupling while at the same time induces large changes in oscillator strength, increasing the radiative decay rate. Thus, the rISC and radiative decay rates will change in step with the different vibrational motions of the molecule, i.e., dynamic oscillations between high rISC and then high radiative decay, taking the two conformers having very different rates as well defined fixed high and low points. One can think of this as the molecule oscillating between high rISC rate and high radiative decay rate configurations. This is dynamic photophysics on vibrational time scales.

In zeonex films, we clearly observe a transition from DF to phosphorescence between 50 and 400 ms, accompanied by a 50–75 meV red shift in emission but no change in the emission band shape. From comparison to our previous measurements of the phosphorescence from triazene^14^ and the phosphorescence measured at 900 ms, we estimate that the phosphorescence in **TpAT-tFFO** is *ca.* 60–80 meV lower in energy than the triazene acceptor. Calculations also consistently show that the first LE triplet state, T_3_ (of the triaxene acceptor), is energetically above T_1_, T_2_, and ^3^CT states and that the T_3_ state emission band should have the vibronic structure (Figure S23). Thus, the observed red shift from 50 to 400 ms in the spectra is not consistent with phosphorescence from the T_3_ LE state. The calculated radiative rate constant for the T_3_ phosphorescence is an order of 1 s^–1^, whereas for the CT triplet states, T_1_ and T_2_, the decay rate is even slower, *ca.* 10^–1^ s^–1^ (in agreement with our visual observation of phosphorescence emission beyond 10 s). We are able to observe phosphorescence at such long times, indicating that whereas the ISC mechanism is still active at 20 K, rISC has been greatly slowed down, leaving a high CT triplet population that can decay radiatively. Additionally, in mCBP, we observe the long-lived phosphorescence at 80 K, and in zeonex, the phosphorescence is quenched at 80 K (Figure S21). This suggests that some molecular motion/reorganization present at 80 K in zeonex switches on TADF, whereas in the small molecule hosts with tight packing, this degree of freedom is hindered such that we still observe the phosphorescence at 80 K. Taken all together, especially the red shift in the spectra, the lack of vibronic structure, and the exceedingly long lifetime of the longest emission, we conclude that the weight of experimental and theoretical evidence supports this phosphorescence to come from a triplet charge transfer state (^3^CT). This represents the strongest evidence so far for radiative decay of a triplet CT excited state.

In **TpAT-tFFO**, all the lowest energy singlet and triplet states are extremely close to each other energetically, so energy gaps are always very small, effectively nearly independent of temperature. In this case, the rISC rate must then depend far more on the dynamic vibrational coupling mechanisms, and vibrational adiabatic coupling only changes the state populations. As the average rISC rate slows down, a larger CT triplet population will build up leading to CT phosphorescence. As the ^3^LE local state is above ^3^CT, the possibility of triplet–triplet annihilation (TTA) through the highly immobile CT triplets contributing to the DF is remote,^33^ compounded by the low concentration of **TpAT-tFFO** molecules in the high triplet energy hosts studied here.

**Scheme 1:**
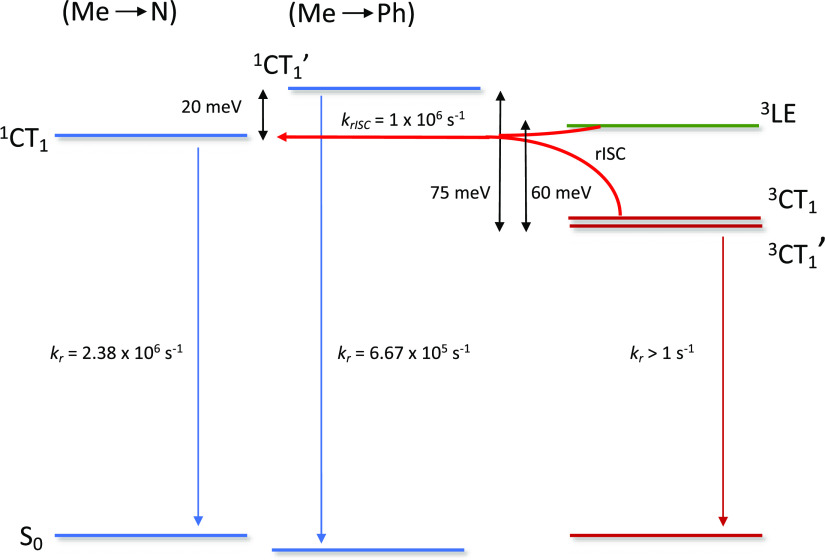
Proposed Energy State Diagram The diagram is for the lowest energy states of the conformers (Me → N)^1^CT_1_ and ^3^CT_1_ and (Me → Ph)^1^CT_1_′ and ^3^CT_1_′, based on the S_0_ (Me → Ph) reference following the nomenclature used in our sister theory paper (Figure S27 of ref (22)). The (Me → N)–(Me → Ph) energy splitting is taken from theoretical estimates, and other energies are the experimentally determined values from the 50 ms delayed fluorescence and 900 ms phosphorescence measured at 20 K. The ^3^LE energy is taken as that previously reported by us for triaxene.^14^ The rISC rate is taken as the fastest rate calculated from the room-temperature delayed fluorescence decay. Radiative decay rates are experimentally determined as described in the text.

From all of our results on the triplet states in **TpAT-tFFO**, we can understand why the TADF is so efficient in this TSCT system. Scheme 1 shows the energy alignment of the three states directly involved in TADF. We see that even though the gap between ^1^CT and ^3^CT is approximately 50–75 meV,^3^LE is close to ^1^CT, which means that they will have a large Franck–Condon overlap, so the rISC (and ISC) step will be very efficient, and most probably the non-adiabatic coupling between ^3^CT and ^3^LE dominates the temperature dependence of rISC. As discussed by Gibson and Penfold,^19^ this configuration of states should give rise to faster and more efficient rISC because the adiabatic coupling is active even at very low temperatures, with low energy torsional modes of D with respect to A greatly contributing to both high rISC and oscillator strength in a dynamic fashion. This is observable in the TSCT **TpAT-tFFO** because the triptycene scaffold holds the D and A units in an average position that ensures very small electron exchange energy such that the small amplitude motions have a large effect on rISC and radiative decay; hence, the triptycene scaffold is extremely important in **TpAT-tFFO** as well.

## Conclusions

This study of the archetypical through-space charge transfer (TSCT) TADF material **TpAT-tFFO** reveals many new facets of the photophysics controlling rISC in TSCT excited states. We identify the role-played by two spatial molecular conformers, energetically separated by 20 meV, that readily interconvert in the ground state but have a large reorganizational energy barrier for interconversion in the excited state. We observe two prompt emission decay times (radiative decay rates of 2.38 × 10^6^ and 6.67 × 10^5^ s^–1^) but no time-dependent change in emission spectra, showing that the species are very close in energy, i.e., the two conformers. We show that ISC is thermally activated, mediated by a vibronic coupling mechanism. It is both efficient and very fast, >10^7^ s^–1^, quenching prompt emission in 30 ns, so that more than 97% of light generated by **TpAT-tFFO** is delayed fluorescence. The DF thus becomes the dominant emission after only 30 ns. Notably, time-resolved measurements show no change in the emission spectral band shape and position from the nanosecond to more than 50 ms. The rISC rate is more strongly thermally activated than ISC, reducing by an order of magnitude more than the ISC rate at low temperature, fully in line with a second-order vibronic coupled SOC mechanism mediating rISC. However, we still observe DF at 20 K in films. The two conformers dictate delayed emission at long times and low temperatures as the population of the slower rISC rate conformer increases as the temperature reduces because interconversion between conformers (over the large excited state reorganizational energy barrier) reduces, meaning that relatively more of this “slow” conformer persists giving rise to a growing long lifetime non-exponentially decaying DF tail. This DF tail is not due to disorder but a consequence of the energetics of conformer interconversion. This gives new insight into these often-seen long DF tails.

We discover that vibrational torsion motions of the D relative to the A units allow the molecule to dynamically access spatial and orientational D–A configurations, oscillation between high *k*_rISC_ and high radiative decay rates at vibrational frequencies, achieving both high overall PLQY and high rISC rates. Our theoretical models show us that the electron exchange energy (or singlet–triplet energy gap) and SOC are very sensitive to changes in the D–A spatial overlap. Thus, even small amplitude vibrational motions of the D and A relative to each other cause large dynamic variations of these rates on the vibrational time scale.

We observe host-independent, temperature-dependent rISC, with an activation energy of 17 meV (i.e., 140 cm^–1^), and freezing-out these low energy torsional vibrational modes reduces rISC, by an order of magnitude more than the corresponding drop in ISC at low temperature. This dynamic behavior is a key difference in **TpAT-tFFO** (and we propose in other TSCT molecules) because the D and A units can move easily with respect to each other in space via many fast vibrational modes, with SOC being very sensitive to this motion, i.e., especially the face-to-face D–A overlap. Meanwhile, through-bond TADF systems with D and A directly bridged by a C–N bond for example have more limited relative motion, mainly slow torsional vibrational degrees of freedom. Finally, all calculations show that in **TpAT-tFFO**, the ^3^CT triplet states are energetically lower than the lowest energy local ^3^LE triplet state, in agreement with the triplet energy of triazene, and given that we observe no change in the emission spectral band shape, but a 50–75 meV red shift between DF at 50 ms and phosphorescence beyond 400 ms (lasting >10 s), we conclude that phosphorescence in **TpAT-tFFO** comes from radiative decay of a charge transfer ^3^CT triplet state. The relative energy gaps determined from our triplet state measurements and calculations, as shown in Scheme 1, indicate that ^1^CT and ^3^LE are close in energy and this is why ISC and rISC are both fast and highly efficient even at low temperature, confirming that **TpAT-tFFO** really defines the state of the art in TADF materials (Figure S8).

**TpAT-tFFO** is an excellent TADF emitter, yielding very high-performance devices. We believe this because in TSCT **TpAT-tFFO**, the D and A can move in three dimensions with respect to each other while still being held in near ideal low electron exchange energy configuration by the triptycene scaffold. For an exciplex TADF system, D–A configuration is random in the bulk in comparison. Vibrational motions allow the **TpAT-tFFO** molecules to dynamically sample different D–A spatial orientations, which have either very high rISC rates or very high radiative decay rates, so that on average, **TpAT-tFFO** is optimized both for the fastest rISC and highest radiative decay. This is an intrinsic property of the dynamic vibrational motion of the molecule, which indicates that the complete rigidity in these through-space TADF emitters would be detrimental. Instead, this degree of freedom enables the molecule to hunt through different conformations to “self-optimize” itself for TADF efficiency. In this respect, **TpAT-tFFO** offers a template for the ideal TADF molecules.

## Supporting Information Available

Steady-state and time-resolved optical measurements, non-adiabatic energy gap estimate, theoretical vibrational couple modes and rate constants, and tables for different hosts (PDF)
