# Supplementary material for: Conformational, Host, and Vibrational Effects Giving Rise to Dynamic TADF Behavior in the Through-Space Charge Transfer, Triptycene Bridged Acridine-Triazine Donor Acceptor TADF Molecule TpAT-tFFO
Source: J Phys Chem C Nanomater Interfaces. 2023 Apr 27;127(18):8607–17. doi: 10.1021/acs.jpcc.2c07529 (PMC10184167; doi:10.1021/acs.jpcc.2c07529)
Supplement: Supplementary file 1 — jp2c07529_si_001.pdf [file jp2c07529_si_001.pdf]

Conformational, Host and Vibrational Effects Giving Rise to Dynamic TADF Behaviour in the Through-Space Charge Transfer, Triptycene Bridged Acridine-Triazine Donor Acceptor TADF Molecule TpAT-tFFO

Hector Miranda-Salinas[a], Angela Rodriguez-Serrano[b], Jeremy Kaminski[b], Fabian Dinkelbach[b], Nakagawa Hiromichi [c], Yu Kusakabe[c], Hironori Kaji[c], Christel M. Marian[b] and Andrew Monkman[a,†]

[a]OEM research group, Dept. of Physics, Durham University, Durham, UK, DH1 3LE

[b]Institut für Theoretische Chemie und Computerchemie, Heinrich-Heine-Universität Düsseldorf, Universitätsstraße 1, D-40225 Düsseldorf, Germany

[c] Institute for Chemical Research Kyoto University, Uji, Kyoto 611-0011, Japan

† a.p.monkman@durham.ac.uk

Supplementary information.

Solution state data

Excitation profiles

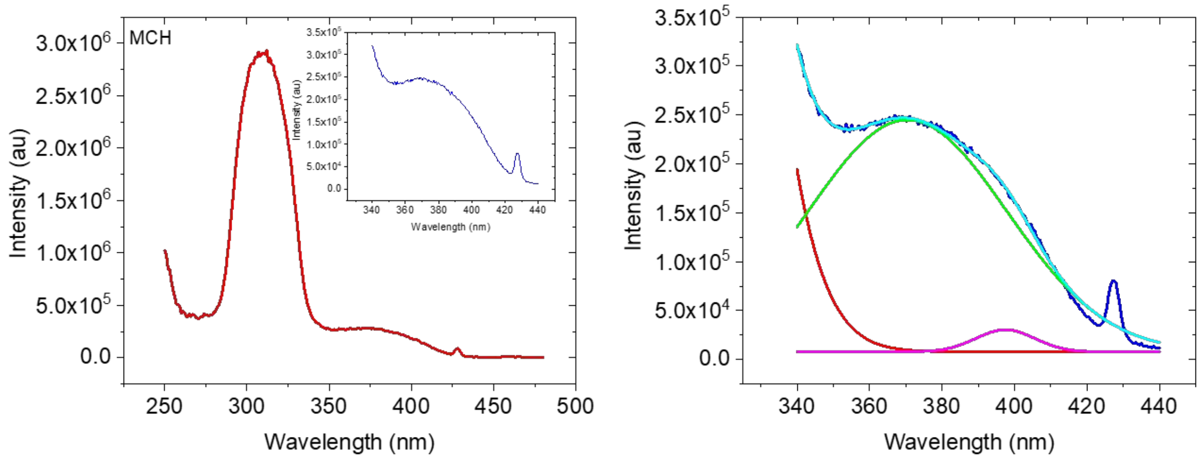

Model	Gauss		
Equation	y=y0 + (A/(w*sqrt(pi/2)))*exp(-2*((x-xc)/w)^2)		
Plot	Peak1(Intensity)	Peak2(Intensity)	Peak3(Intensity)
y0	7862.25392 ± 4687.13599	7862.25392 ± 4687.13599	7862.25392 ± 4687.13599
xc	293.01815 ± 113.59812	370.46715 ± 3.22124	397.45612 ± 1.359
w	40.50936 ± 49.35318	54.80797 ± 3.80708	16.2968 ± 3.80701
A	1.39169E8 ± 1.09134E9	1.62873E7 ± 1708902.65174	460446.69821 ± 225420.83745
Reduced Chi-Sqr	7.29062E7		
R-Square (COD)	0.9913		
Adj. R-Square	0.99089		

Figure S1. a) Excitation spectrum of TpAT-tFFO dissolved in MCH, inset shows well resolved direct CT absorption below the strong ππ* transition, and b) close inspection of the 340 – 420 nm band, monitored emission wavelength 470 nm.

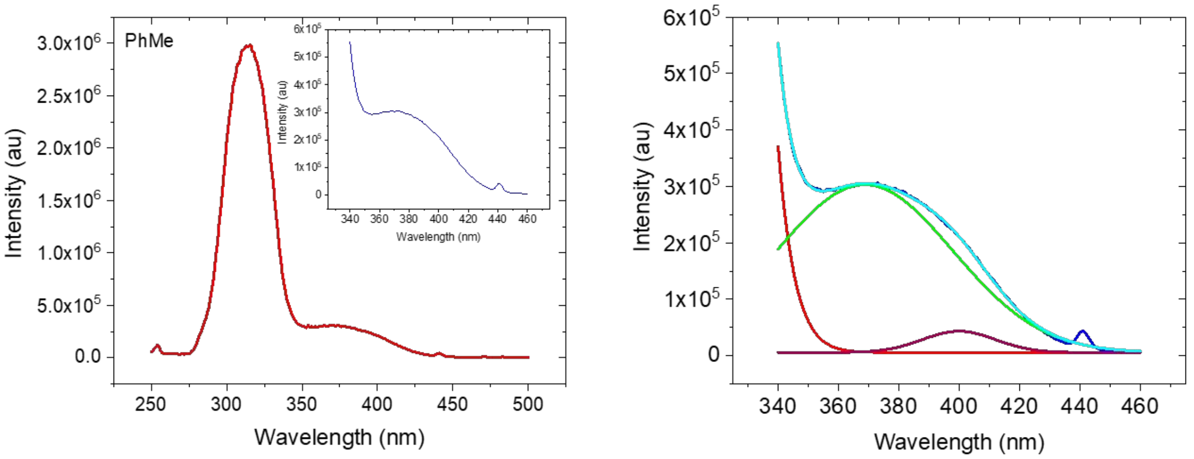

Model	Gauss		
Equation	y=y0 + (A/(w*sqrt(pi/2)))*exp(-2*((x-xc)/w)^2)		
Plot	Peak1(Intensity)	Peak2(Intensity)	Peak3(Intensity)
y0	5759.77753 ± 1822.34936	5759.77753 ± 1822.34936	5759.77753 ± 1822.34936
xc	261.91885 ± 127.1533	368.71422 ± 1.85728	399.88372 ± 1.21882
w	42.03195 ± 34.11045	58.33925 ± 1.71277	24.2438 ± 3.21309
A	1.91113E10 ± 2.31942E11	2.17442E7 ± 767161.41734	1144985.29435 ± 399134.74698
Reduced Chi-Sqr	2.17034E7		
R-Square (COD)	0.99886		
Adj. R-Square	0.99877		

Figure S2. a) Excitation spectrum of TpAT-tFFO dissolved in PhMe and b) close inspection of the 340 – 420 nm band, monitored emission wavelength 510 nm.

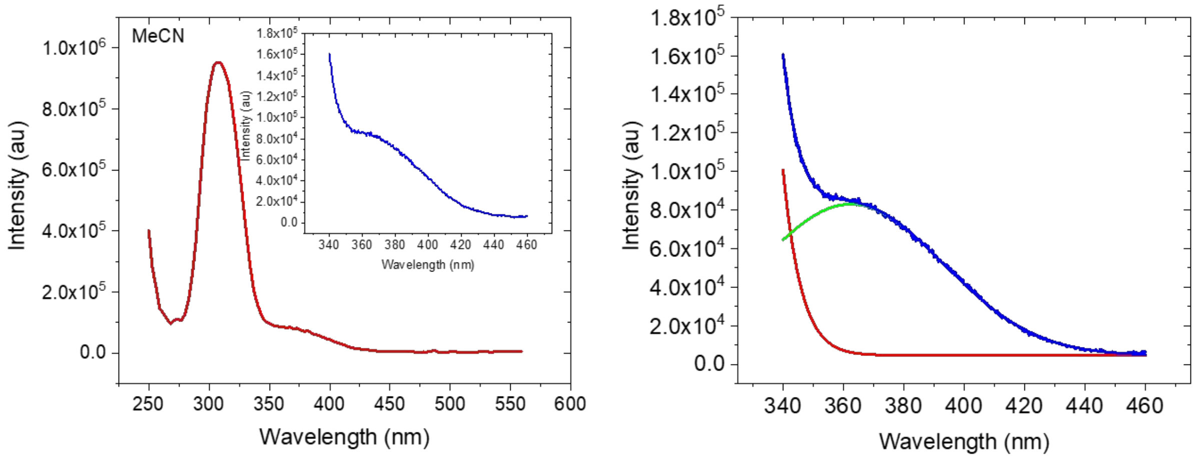

Model	Gauss	
Equation	y=y0 + (A/(w*sqrt(pi/2)))*exp(-2*((x-xc)/w)^2)	
Plot	Peak1(Intensity)	Peak2(Intensity)
y0	4683.12334 ± 186.54336	4683.12334 ± 186.54336
xc	239.3385 ± 81.02958	362.46644 ± 0.60549
w	49.4508 ± 19.71849	61.19907 ± 0.75052
A	2.36669E10 ± 1.69287E11	6008887.43175 ± 125620.10726
Reduced Chi-Sqr	853396.57333	
R-Square (COD)	0.9994	
Adj. R-Square	0.99938	

Figure S3. Excitation spectrum of TpAT-tFFO dissolved in MeCN and b) close inspection of the 340 – 420 nm band, monitored emission wavelength 570 nm.

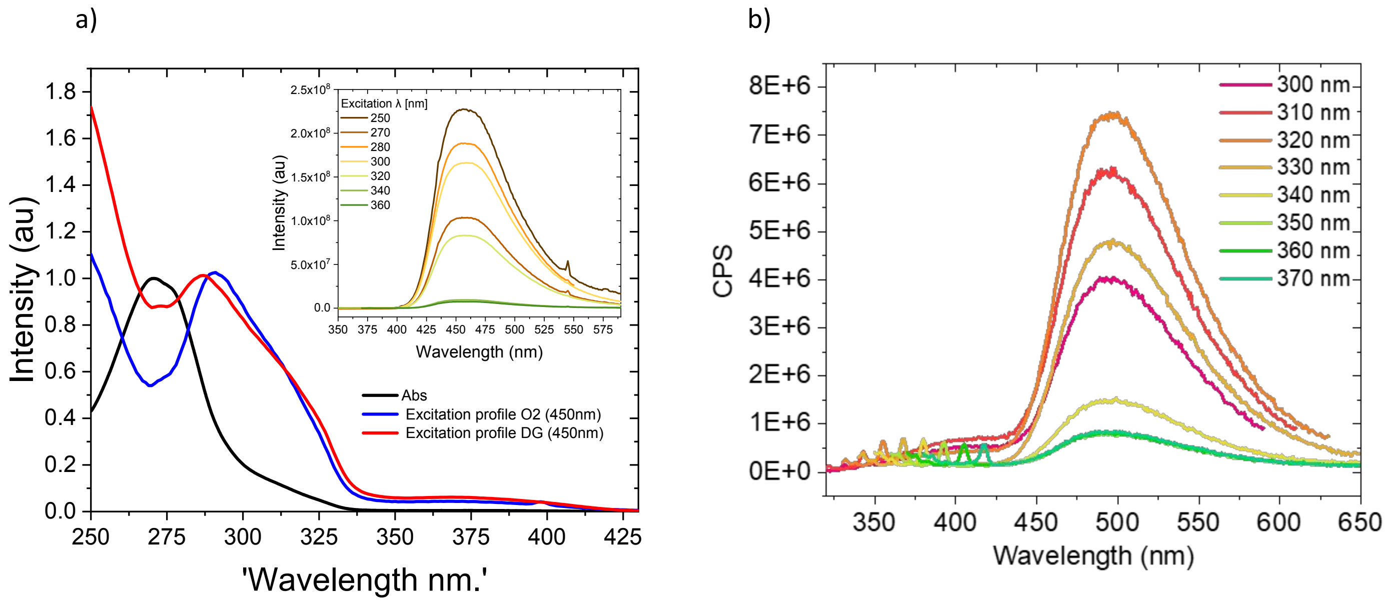

Figure S4. TpAT TFFO emission a) oxygen dependent excitation spectra and b) as a function of excitation wavelength in toluene.

As the S0-S3 (as well as the S0-S1 and S0-S2) transition led to prompt CT emission we can ascribe the primary electron transfer mechanism as hole transfer from A*D. The excitation spectra also indicate that the acceptor transition gives rise to far more CT production even though it has a much weaker extinction coefficient than the donor ππ* absorption, consistent with hole transfer from A*D being the main ET channel.

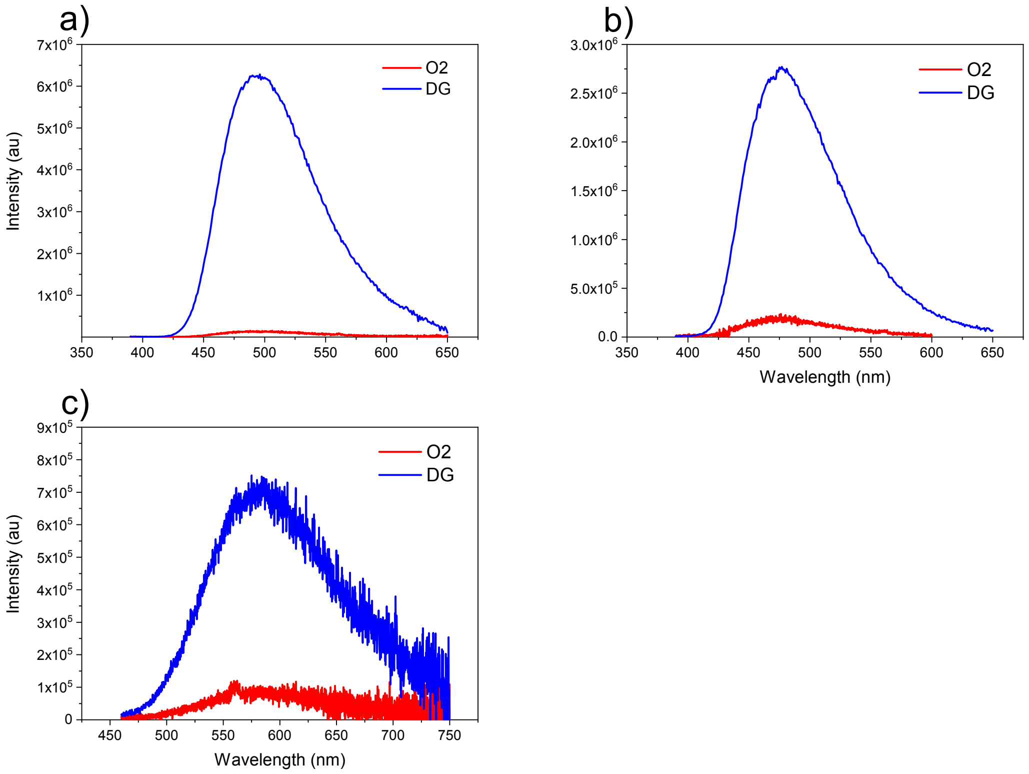

Figure S5. Degassed (blue) and in presence of oxygen (red) steady state emission comparison of TpAT-tFFO at 20 mM inf a) PhMe, b) MCH and c) MeCN.

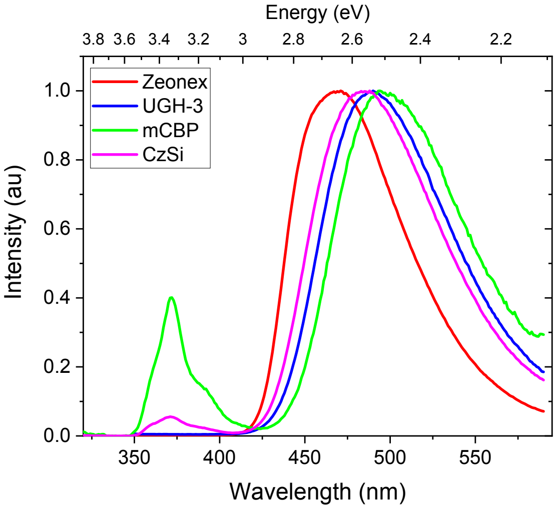

Figure S6. Solid state emission spectra using different hosts Zeonex 1% and the others 10% by weigth.

Toluene data

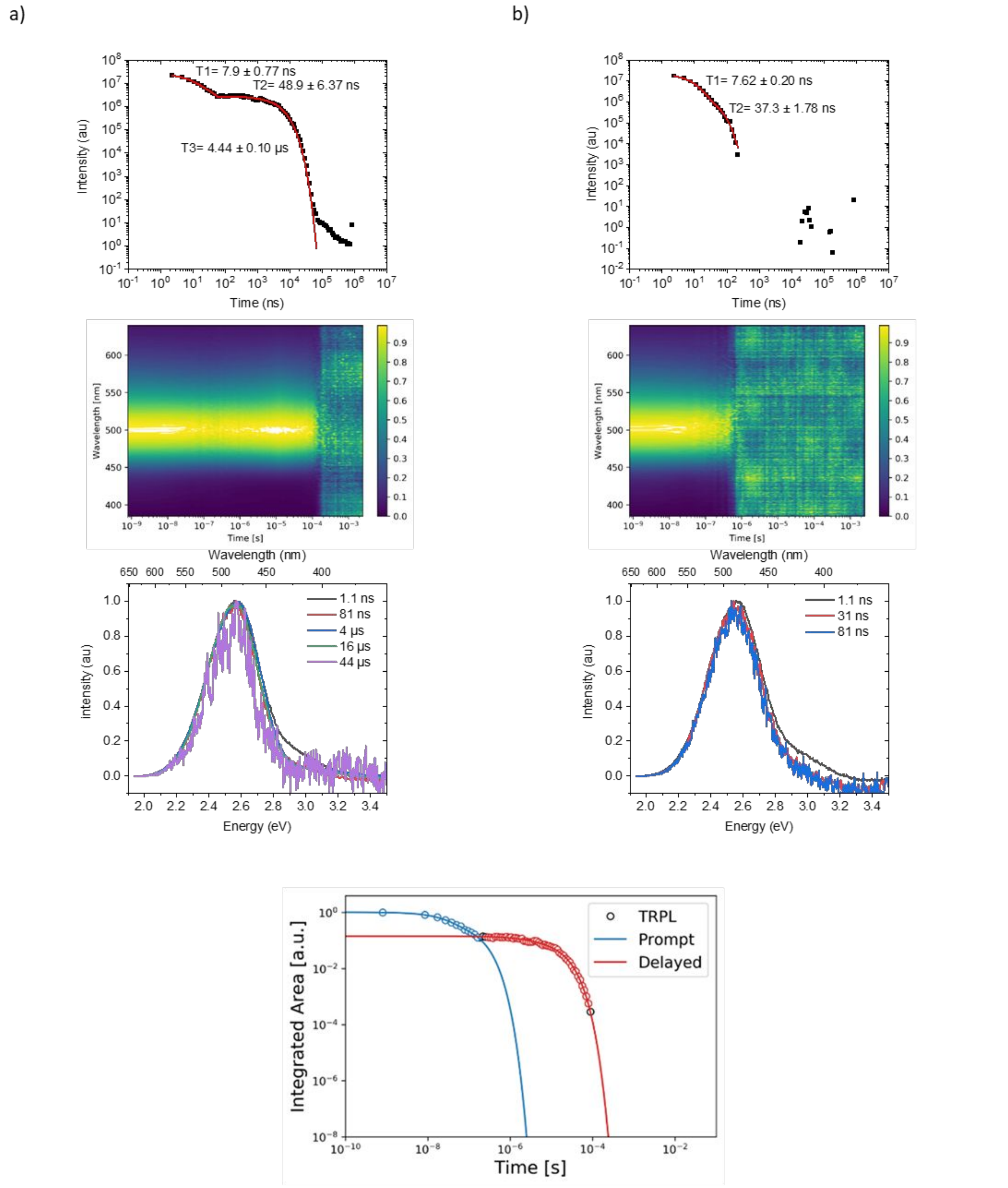

K_F	K_F Error	K_{ISC}	K_{ISC} Error	K_{rISC}	K_{rISC} Error
347006.2	19185.21	12543342	275080.2	3191554	163110.7

Figure S7. TpAT tFFO in PhMe a) degassed and b) oxygenated, time resolved decays, contour plots and their respective frames at different times.

MCH data

a) b)

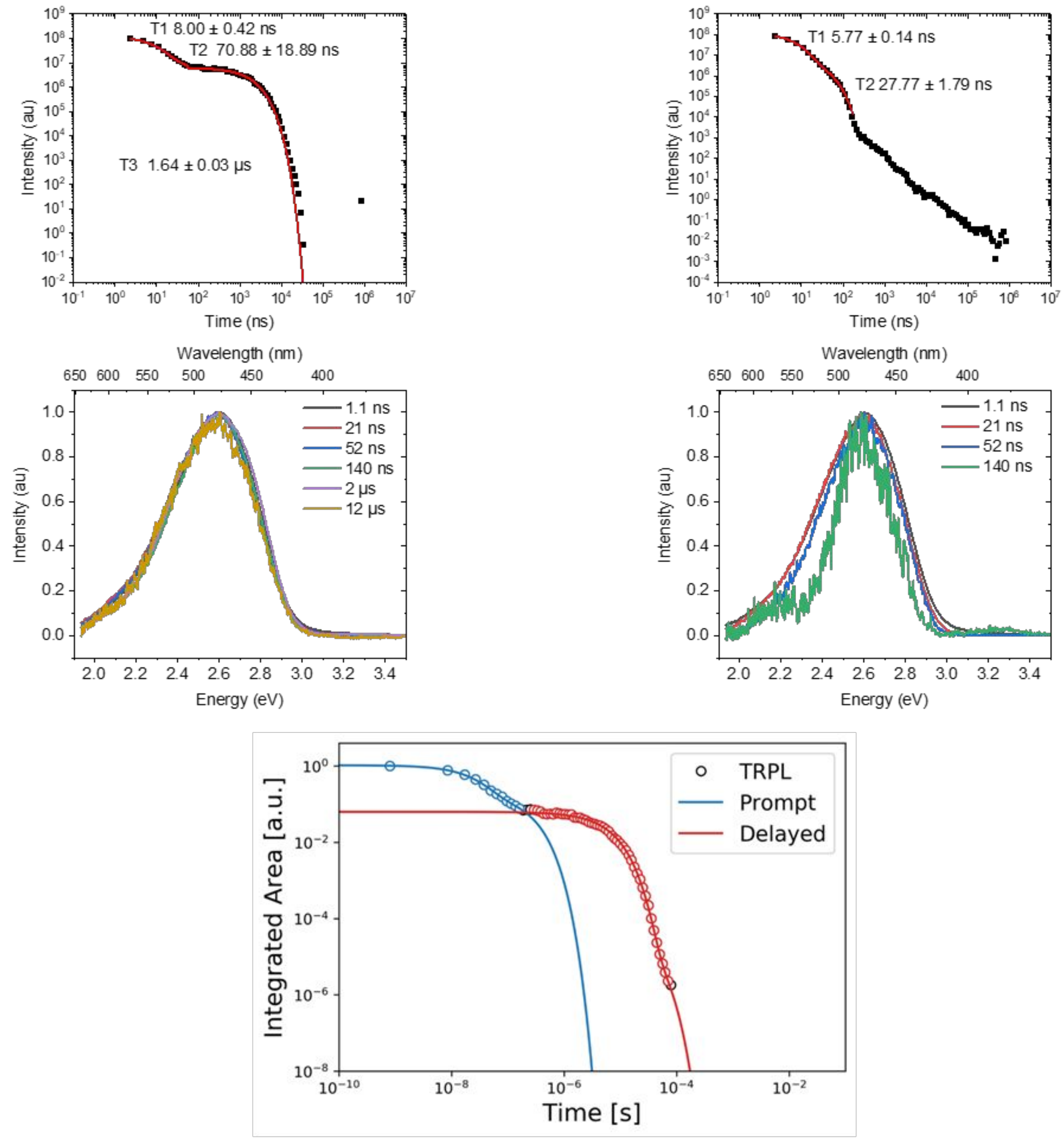

K_F	K_F Error	K_{ISC}	K_{ISC} Error	K_{rISC}	K_{rISC} Error
1905016	148020.8	11318695	28722.48	1129418	101105.4

Figure S8. TpAT TFFO in MCH a) degassed and b) oxygenated, time resolved decays and their respective frames at different times.

MeCN data

a) b)

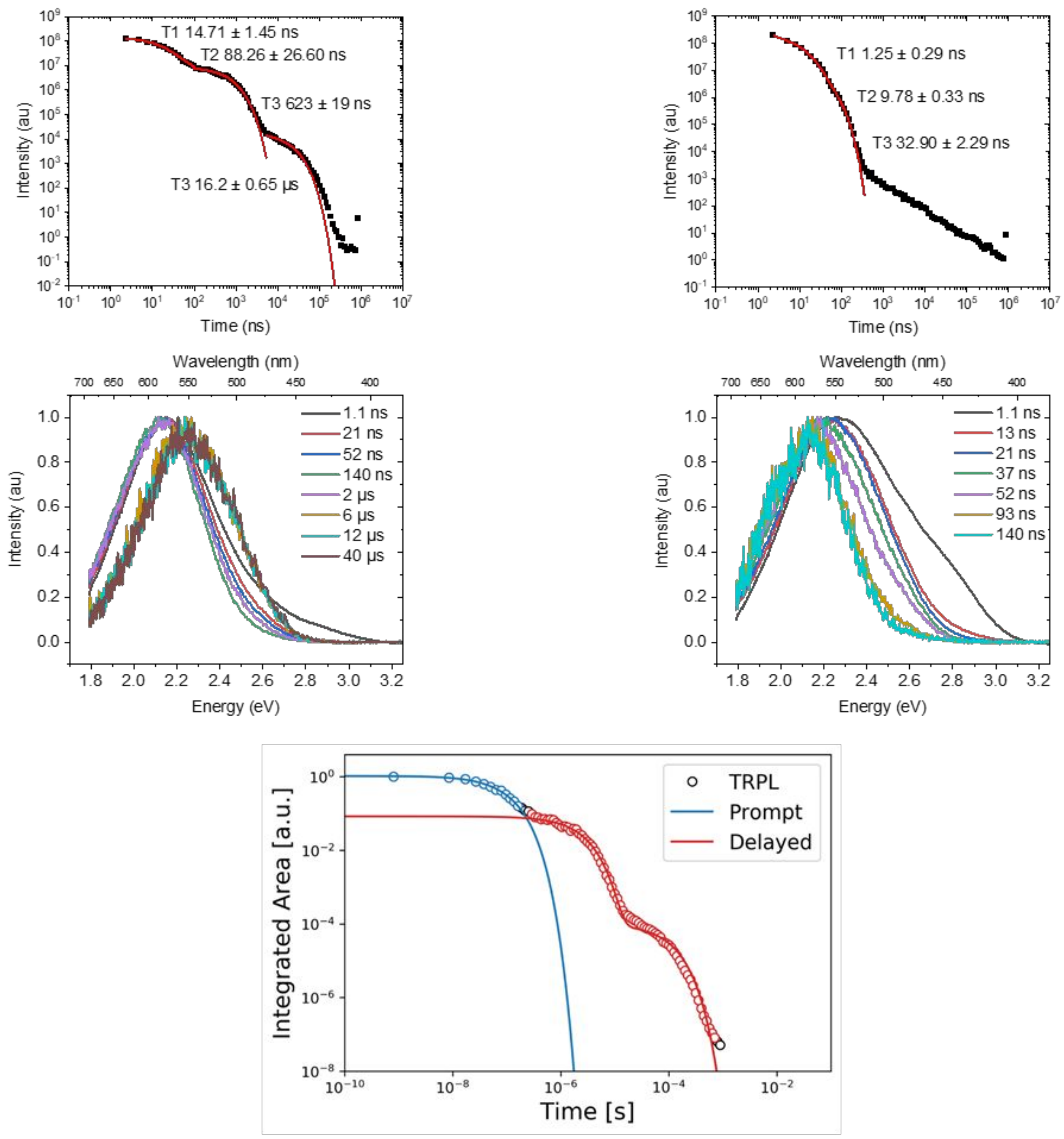

K_F	K_F Error	K_{ISC}	K_{ISC} Error	K_{rISC}	K_{rISC} Error
3689300	161.0247	9814614	641.5399	74871.34	0.000928

Figure S9. TpAT TFFO in MeCN a) degassed and b) oxygenated, time resolved decays and their respective frames at different times.

In MeCN we observe rather more complicated photophysics with lifetimes and spectral components highly modified by the strongly polar environment, figure 4c (main manuscript) and 9a. In aerated solution, figure 9b, we observe multiple prompt decay components and spectral contributions, with large energy splitting of ca. 200 meV. In the first few nanoseconds we observe predominantly a gaussian feature at 550 nm (emission peak, lifetime 9.8 ns) having a high energy knee at 400-475 nm. This knee matches with the CT emission band observed in MCH, having a lifetime estimated at 1.25 ns (very close to our IRF however). These results then suggest that in highly polar MeCN there are aggregates or residual undissolved **TpAT-tFFO** even in low concentration solution. In this case it is difficult to conclude much about the early time photophysics. After some 50 ns, the CT emission has red shifted to 580 nm (onset 2.53 eV) having a lifetime of 33 ns, figure 9b. In degassed solution, figure 4c (main manuscript) and 9a, we observe the same two initial prompt CT components, with slightly longer lifetimes, but delayed CT emission from the low energy species at 580 nm rapidly dominates, clearly indicating that this band is giving a large DF contribution. This initial DF has a lifetime of 625 ns lasting for some 2 μs only. This is very fast for TADF. Over the first 2 μs this highly red shifted feature dominates, but at longer delay times (>2 μs) weak DF emission is observed from the higher energy 550 nm state. This long-time decay

contribution is seen in the kinetics decays as well, figure 9b, having lifetime of 16 µs. If we assume that a mediating local triplet state (for the rISC process) is close to 450 nm, then a CT state at 550 nm would be expected to have rather slow rISC as ΔE_{ST}~0.35 eV, therefore the emission intensity of MeCN is weaker than **TpAT-tFFO** in other solvents, due the lack of efficient rISC and having impact on the overall emission of the molecule. The observed short DF lifetime of the 580 nm species probably reflects a fast non radiative quenching of this state. We note that the PLQY of **TpAT-tFFO** in MeCN is very low (see figure 5c) and supports non-radiative quenching of this state out-competing rISC. We have no clear idea what this 580 nm state is, maybe some highly relaxed CT configuration having efficient TADF through mixing between the S_1 and S_2 triplet CT states mediating rISC instead of the ^{3}LE state being involved. Or possibility this is a photodegradation species given the high fluence of 355 nm used during the time resolved measurements. We do not have enough clear evidence to be conclusive here.

TpAT-tFFO

TCSPC measurements measured in solution.

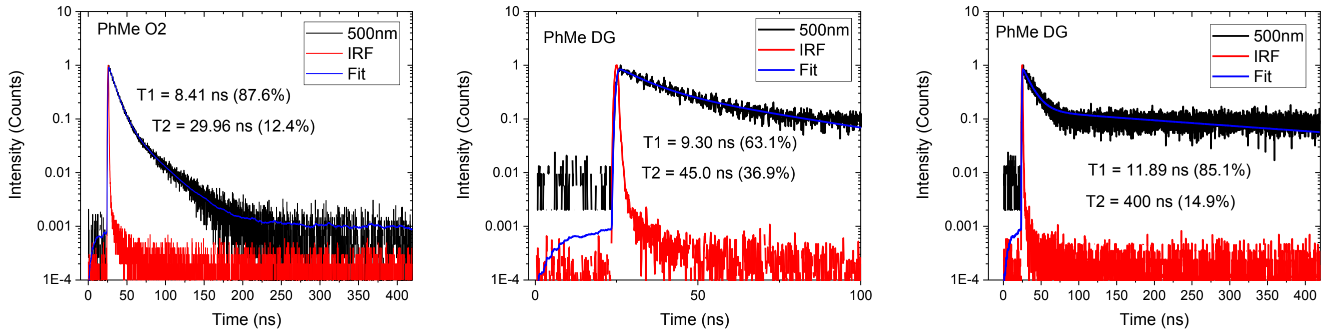

Figure S10. TCSPC decay measurements of TpAT tFFO in PhMe measure in aerated solution a), degassed b) and c) degassed in a longer time range.

Table S1. TCSPC Data for PhMe and MCH.

	**T_1 (ns)**	**T_2 (ns)**
PhMe O_2	8.41 (87.6%)	30 (12.4%)
PhMe DG	9.3 - 12 (63-85%)	>400 (15%)
MCH O_2	6.28 (93.8%)	26.53 (6.2%)
MCH DG	8.8 - 12 (89.9%)	>400 (15%)

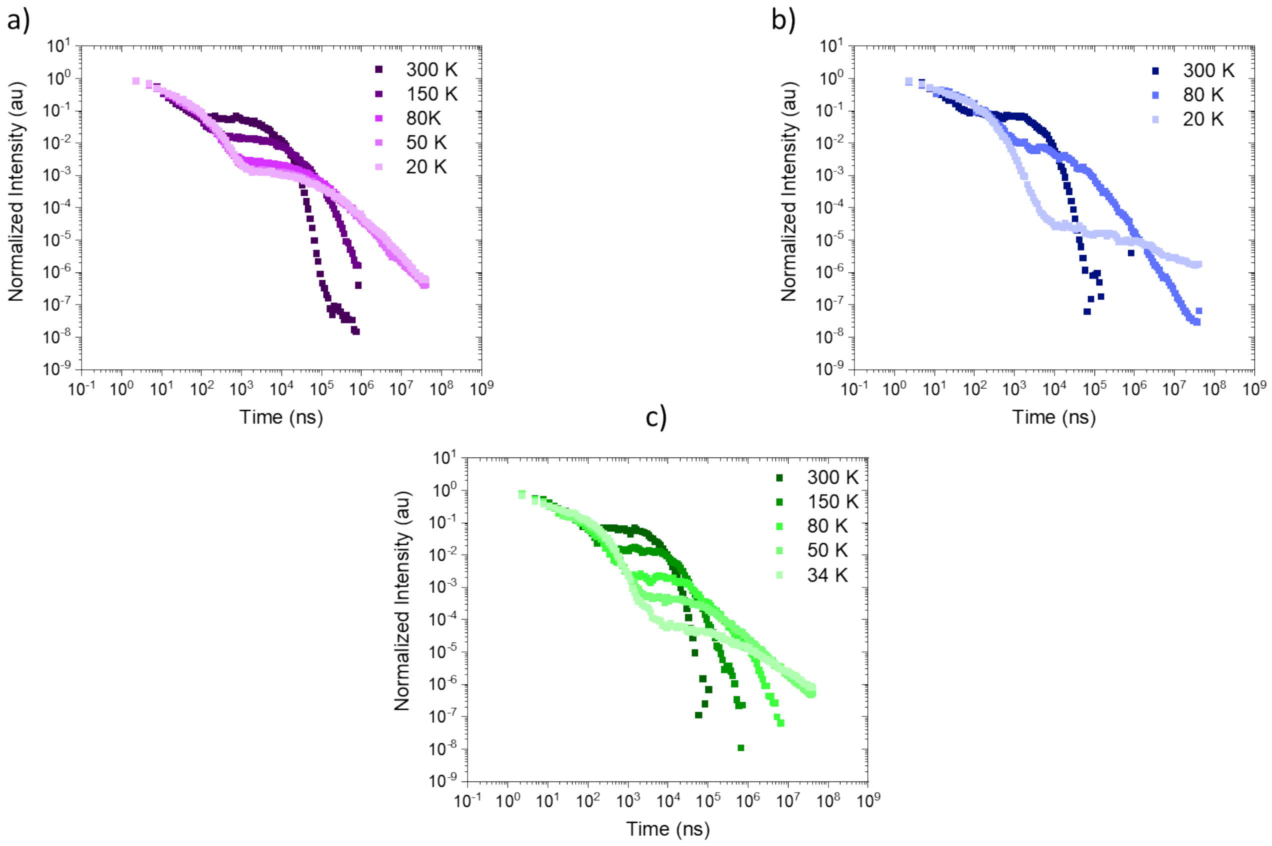

Figure S11 Decays for the time resolved measurements at different temperatures of a) zeonex, b) UGH-3 and c) CzSi.

UGH film data

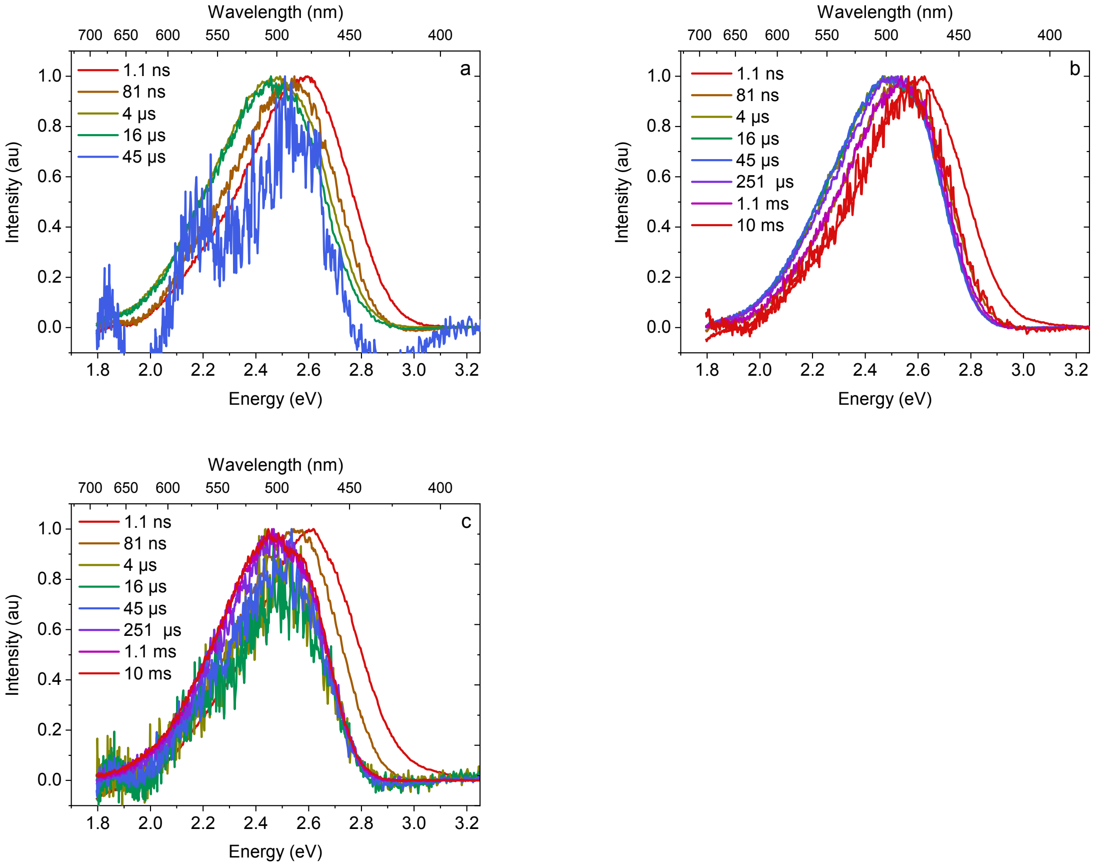

Figure S12. UGH-3 hosted film time resolved different frame times at a) 300 K, b) 80 K and c) 20 K.

a) 300K

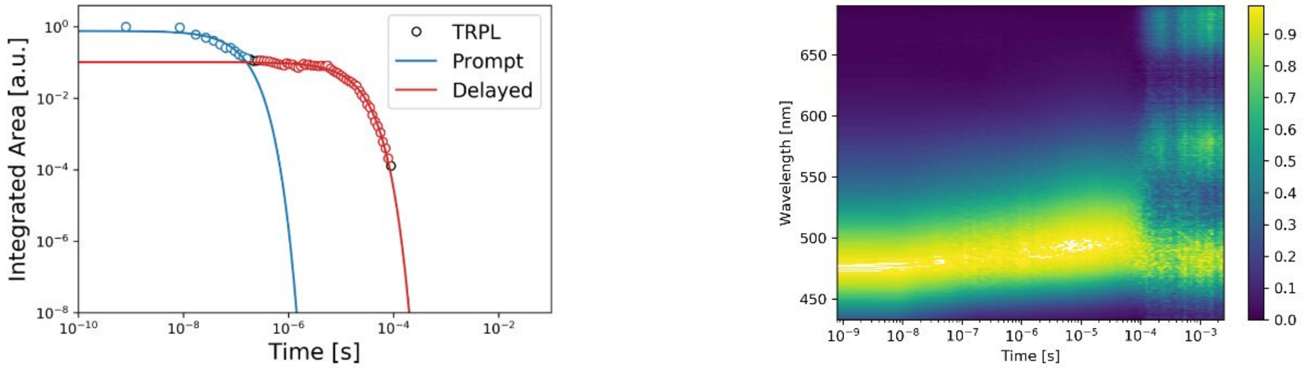

b) 80K

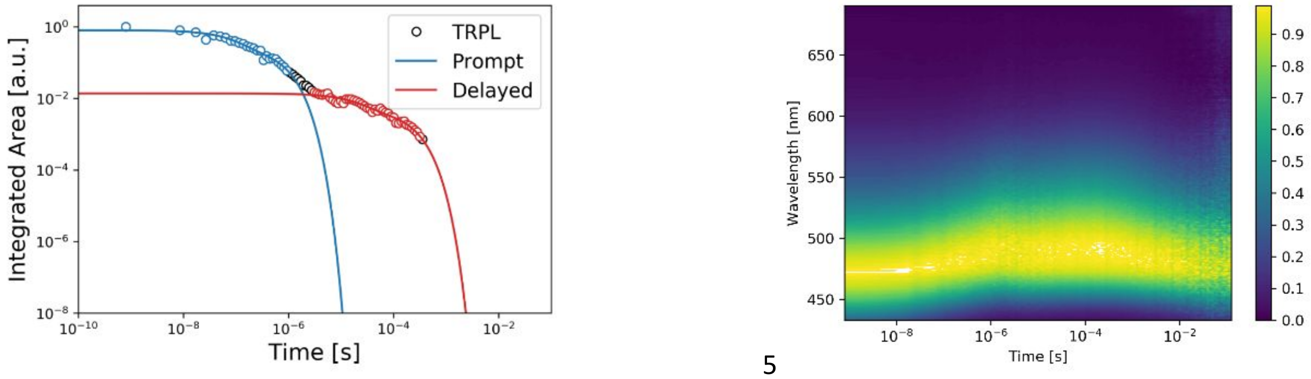

5

c) 20K

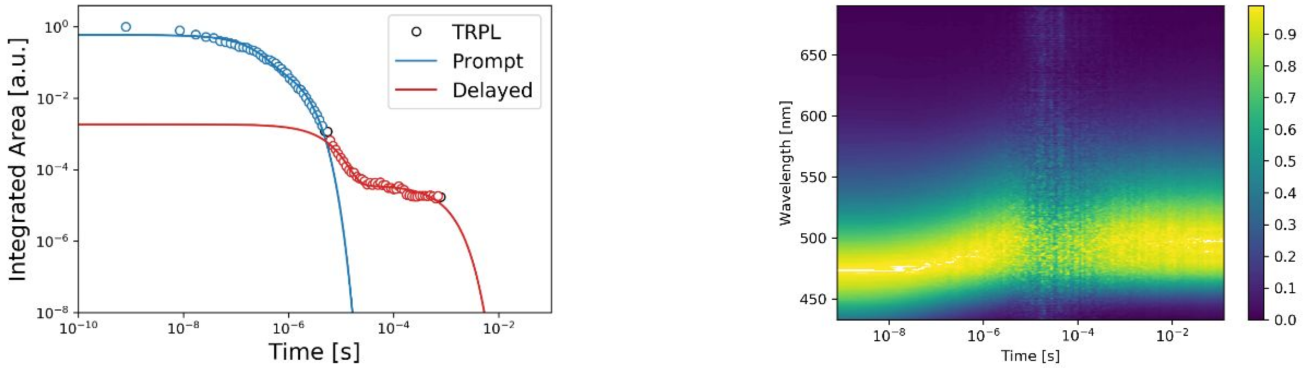

d)

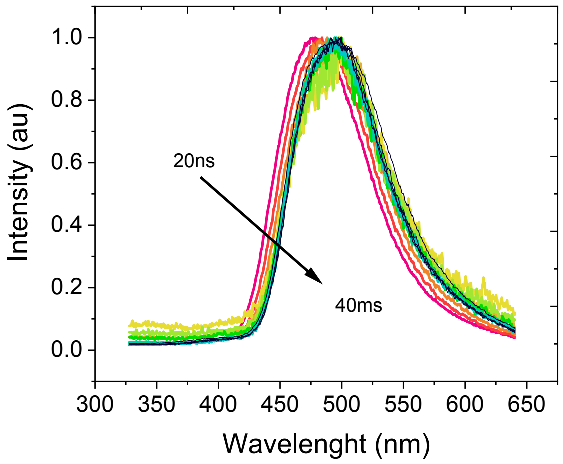

Figure S13. UGH-3 hosted film kinetic decay fitting and contour plot at a) 300 K, b) 80 K and c) 20 K. d) shows detailed time resolved spectra over a wide time scale measured at 80 K.

mCBP film data

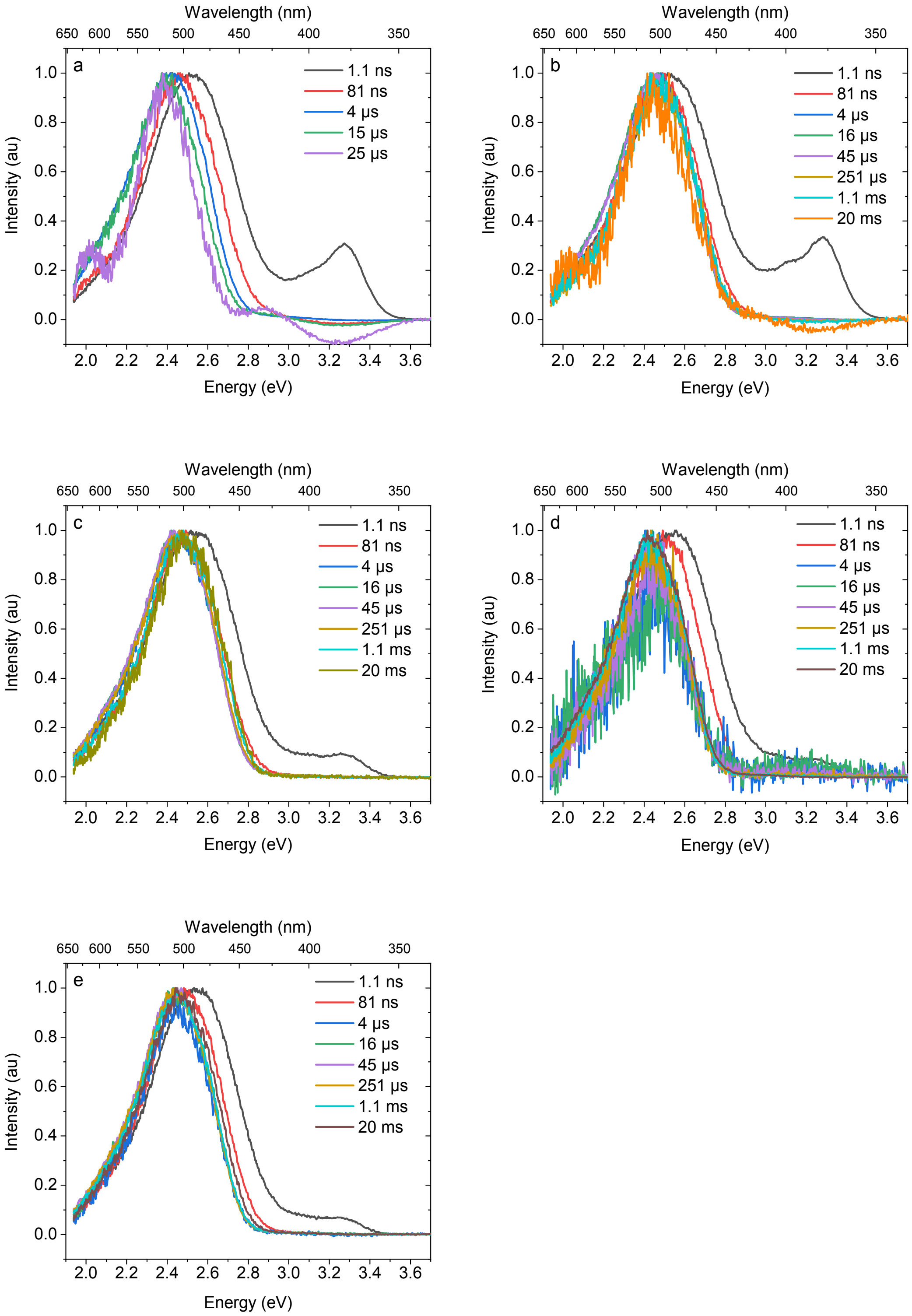

Figure S14. mCBP hosted film time resolved frames at a) 300 K, b) 150 K, c) 80 K, d) 50 K and e) 20 K.

a) 300K

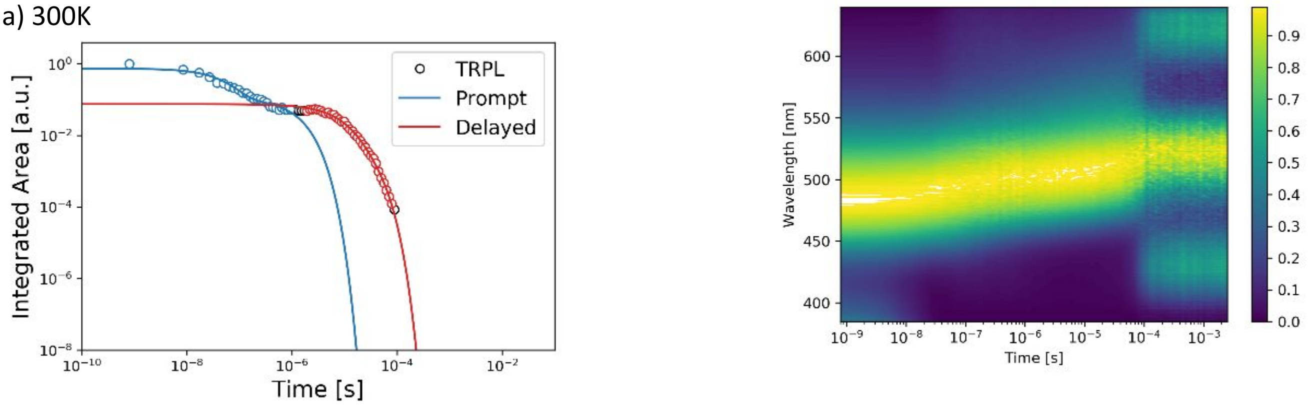

b) 150K

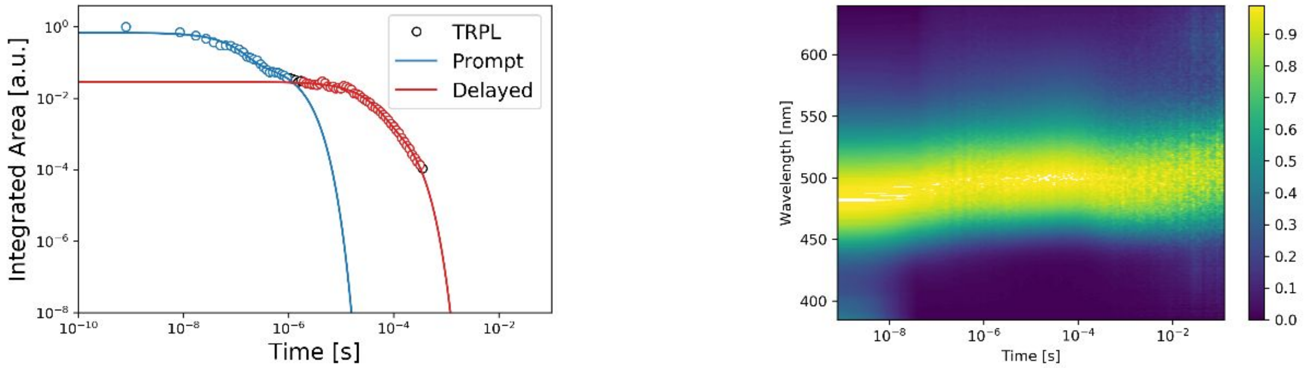

c) 80K

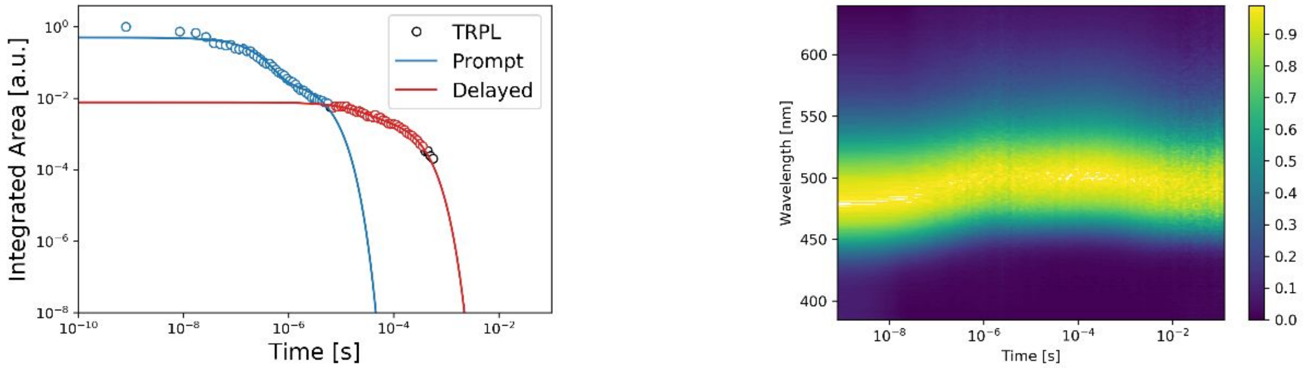

d) 50K

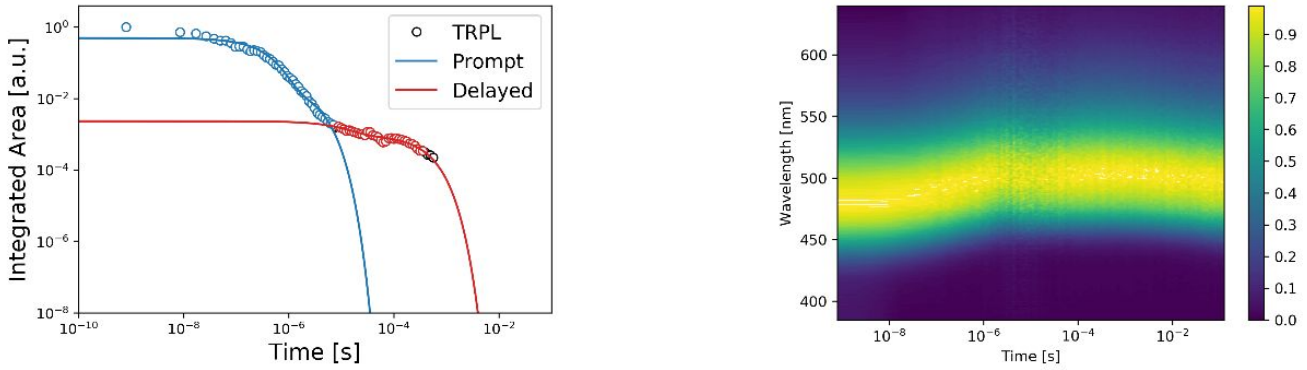

e) 20K

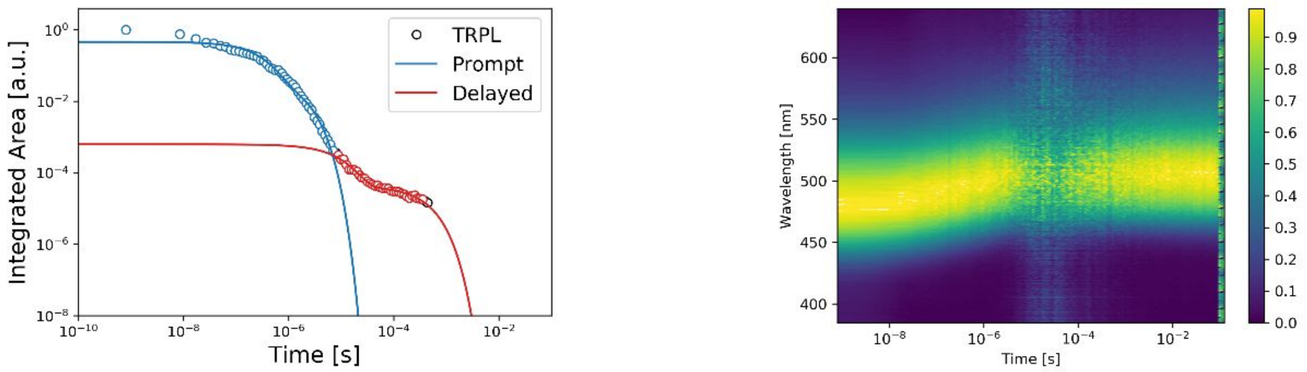

Figure S15. mCBP hosted film kinetic decay fitting and contour plot at a) 300 K, b) 150 K, c) 80 K, d) 50 K and e) 20 K.

CzSi film data

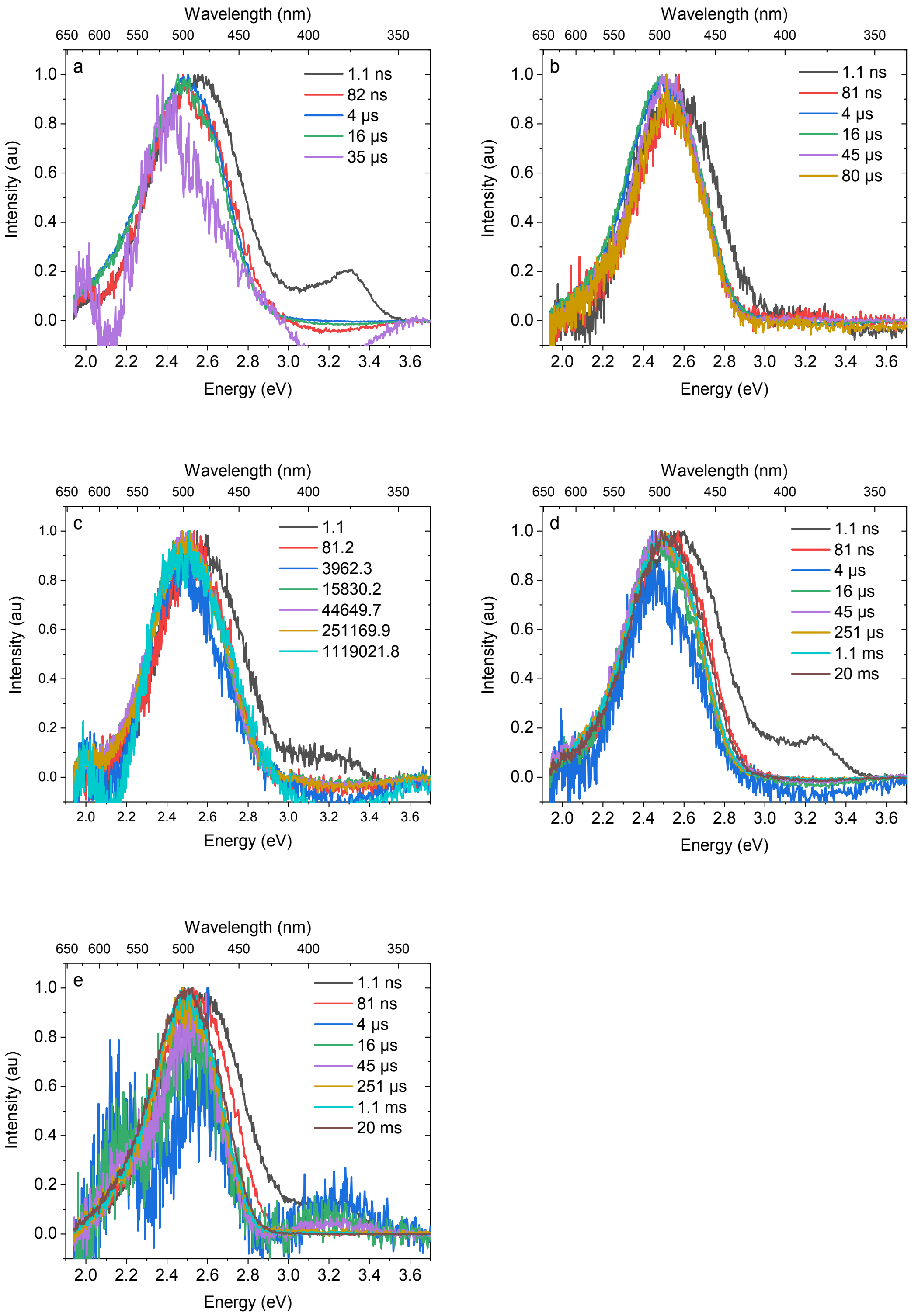

Figure S16. CzSi hosted film time resolved frames at a) 300 K, b) 150 K, c) 80 K, d) 50 K and e) 34 K.

a) 300K

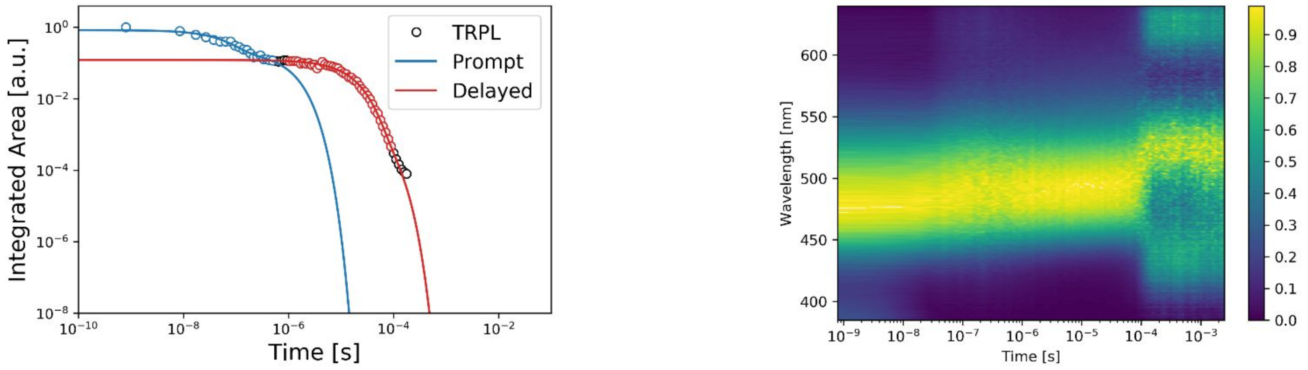

b) 150K

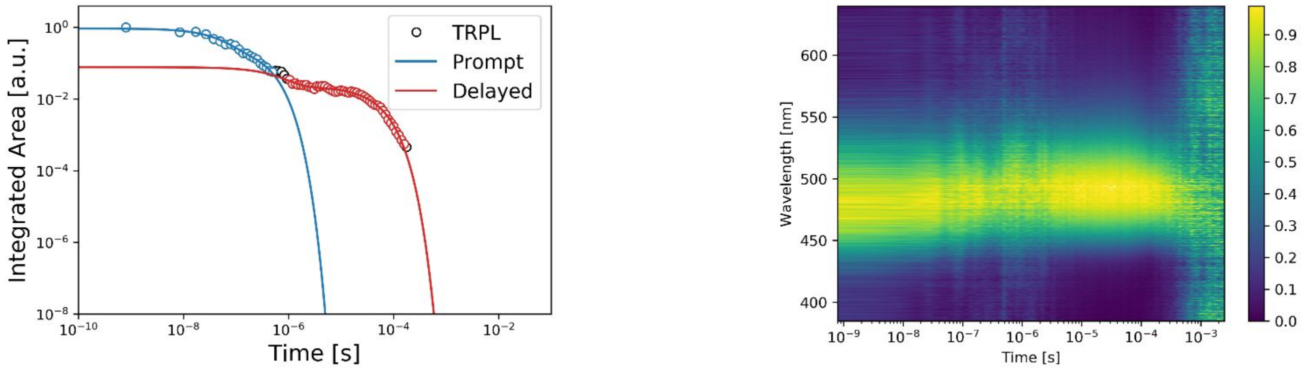

c) 80K

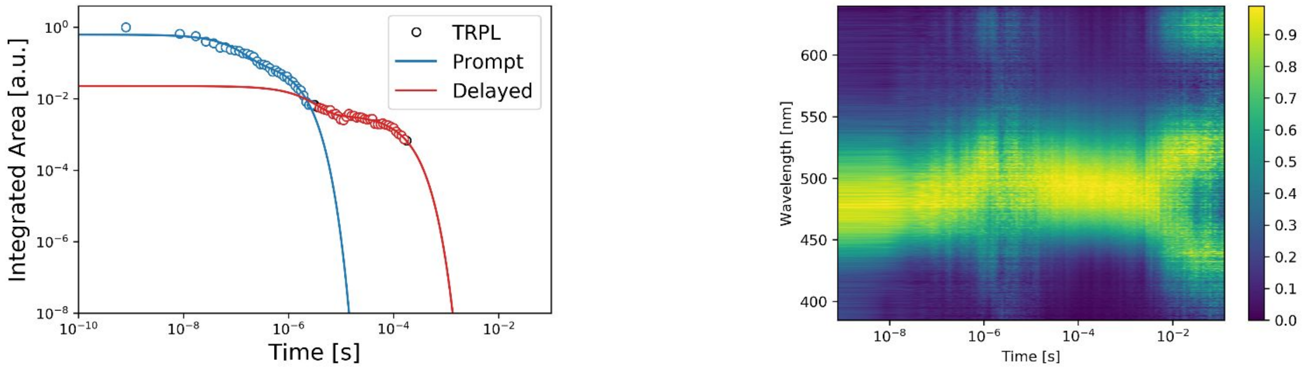

d) 50K

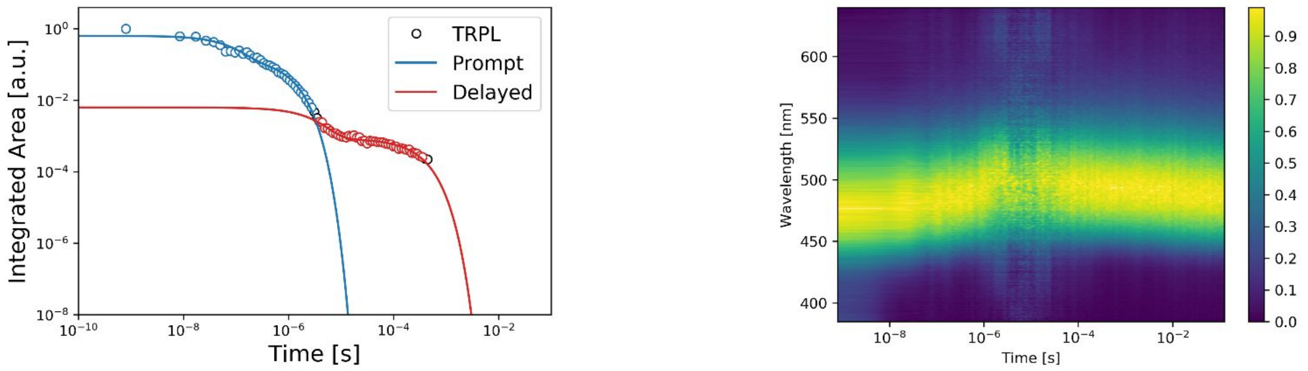

e) 34K

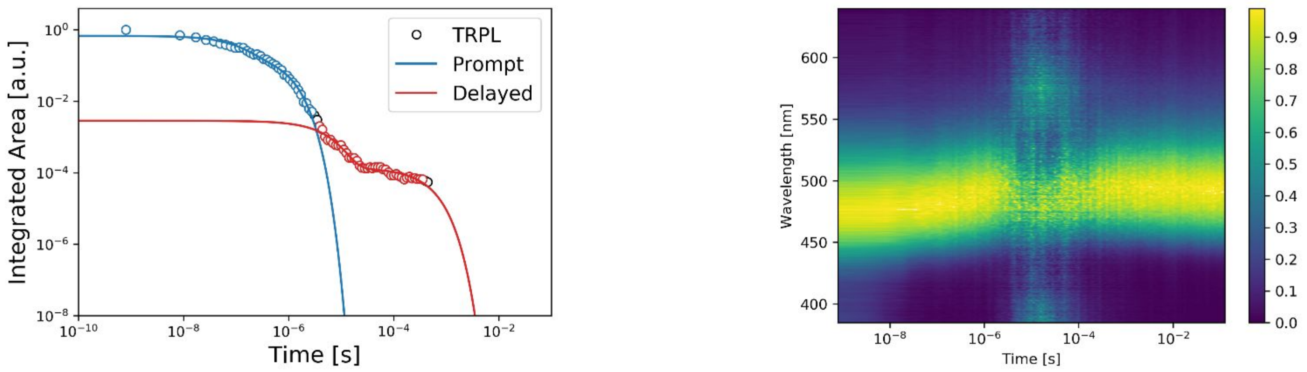

Figure S17 CzSi hosted film kinetic decay fitting and contour plot at a) 300 K, b) 150 K, c) 80 K, d) 50 K and e) 34 K.

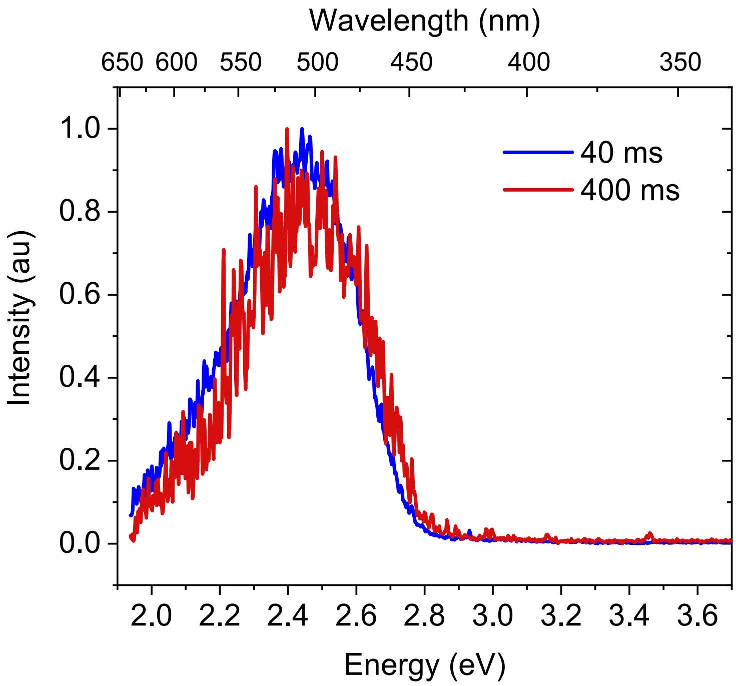

Figure S18 Comparison of emission 40 ms (blue) and 400 ms (red) in the mCBP hosted film at 20 K.

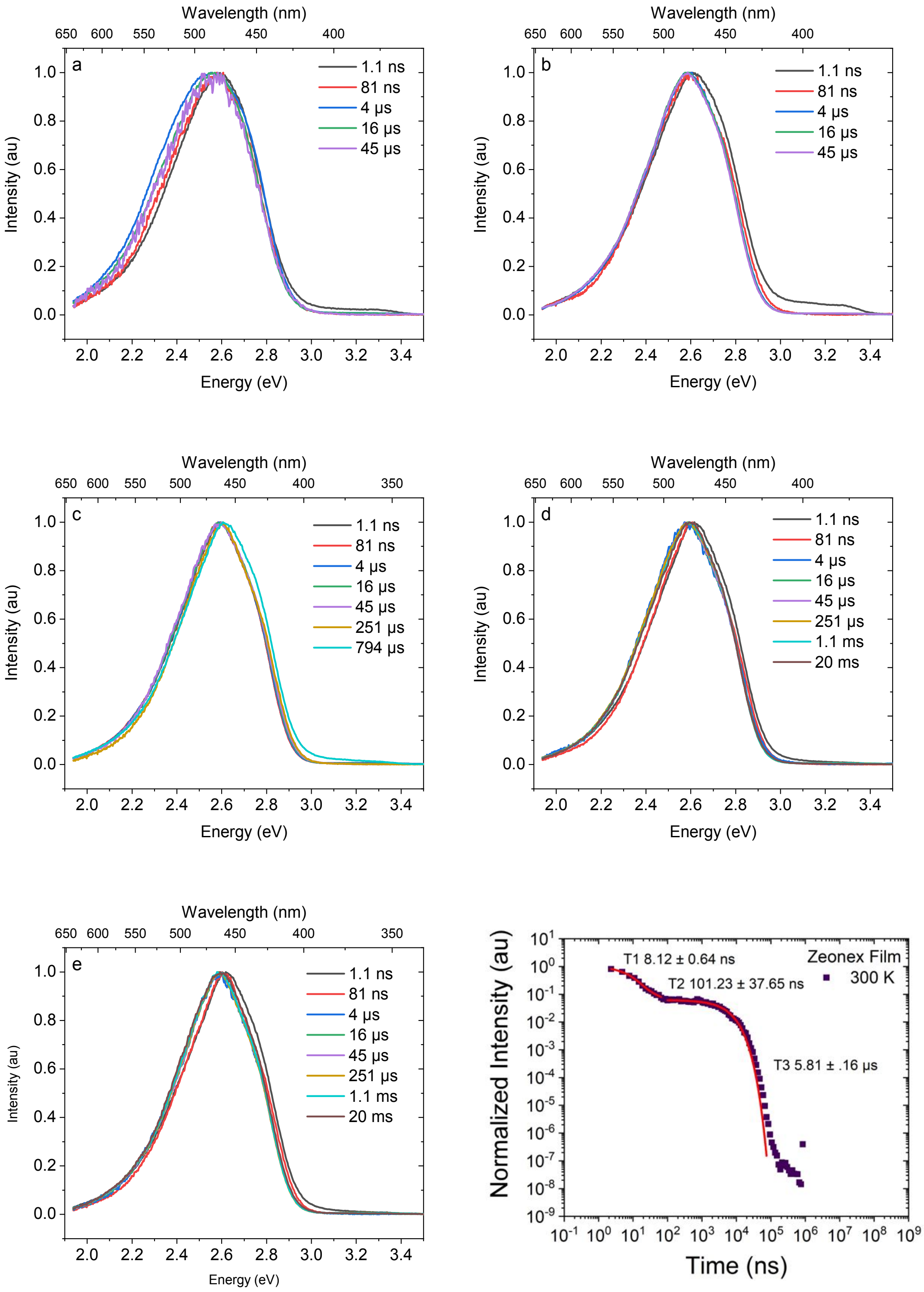

Figure S19. Zeonex hosted film time resolved frames at a) 300 K, b) 150 K, c) 80 K, d) 50 K and e) 20 K. f) shows the exponential fitting of the room temperature decay of emission.

a) 300K

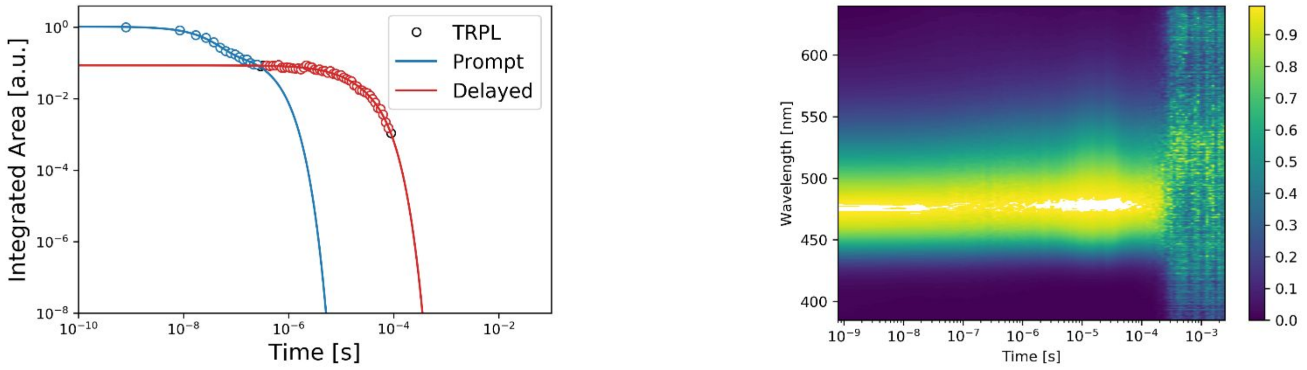

b) 150K

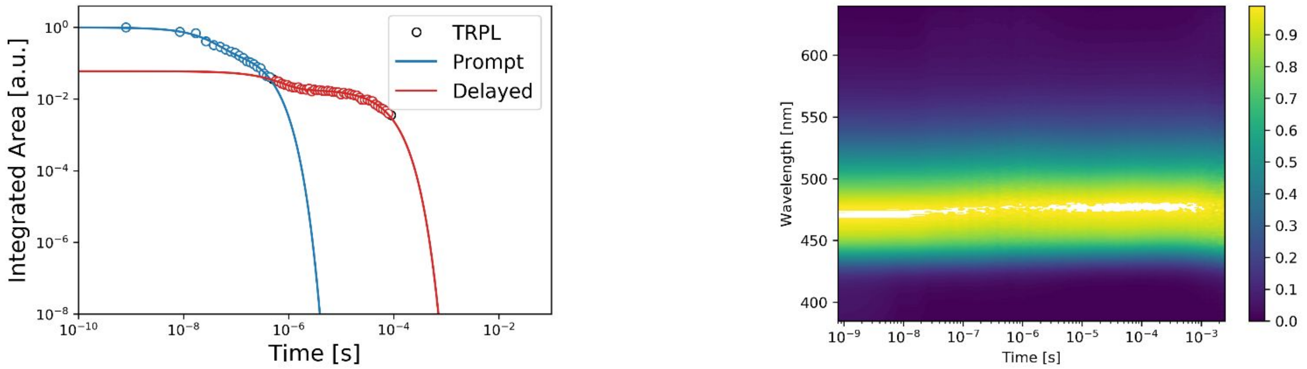

c).80K

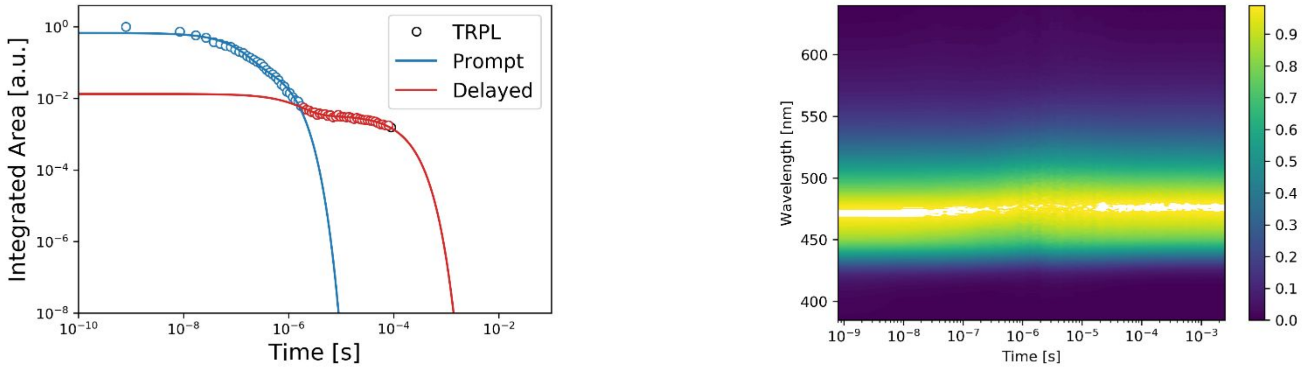

d) 50K

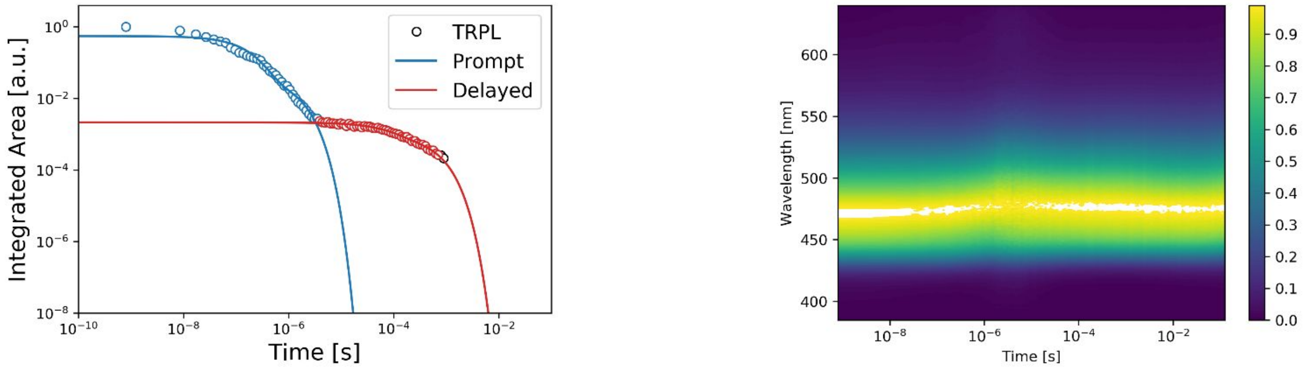

e) 20K

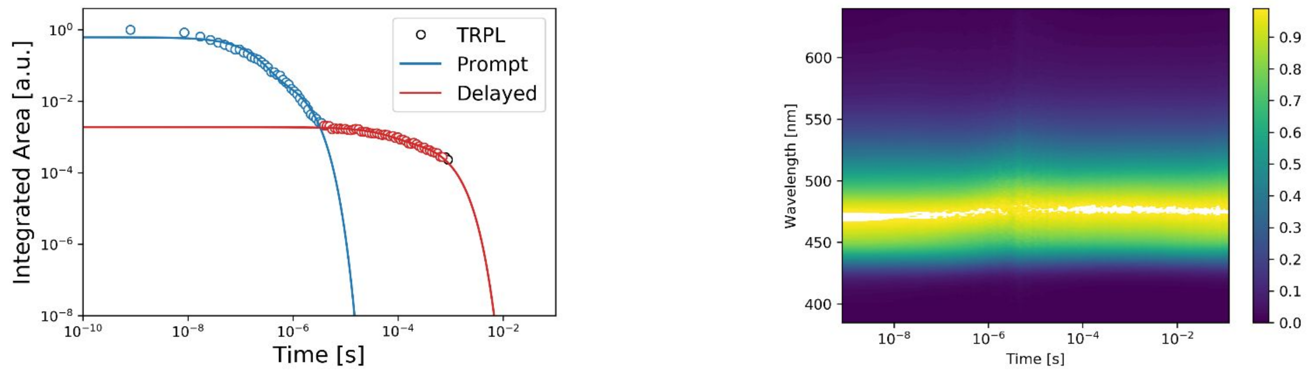

Figure S20 Zeonex hosted film kinetic decay fitting and contour plot at a) 300 K, b) 150 K, c) 80 K, d) 50 K and e) 20 K.

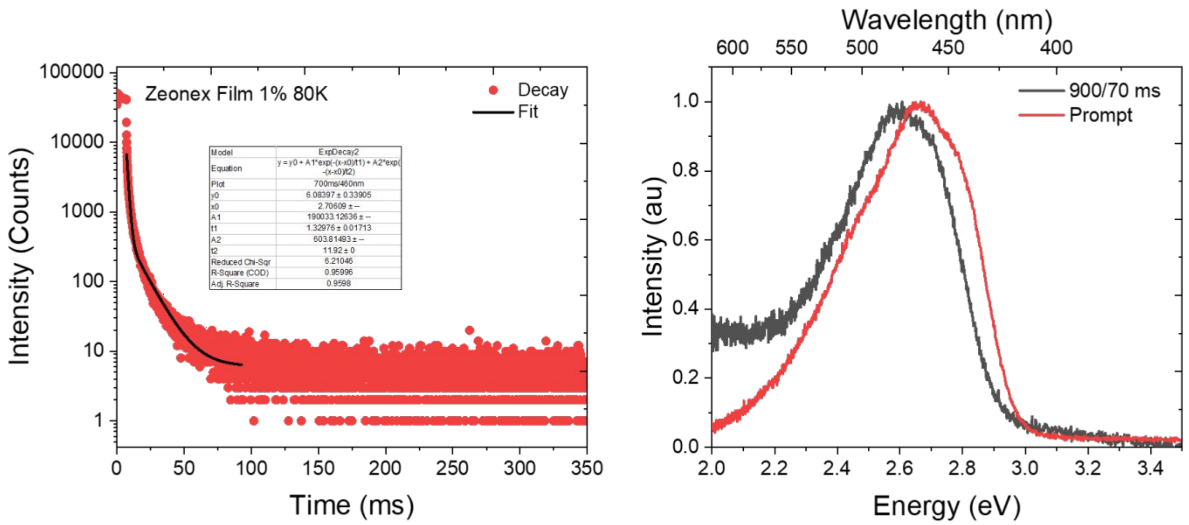

Figure S21 Zeonex hosted film TCSPC decay at 80K and spectra measured at 20K with 900 ms delay time and 70 ms integration time vs prompt emission.

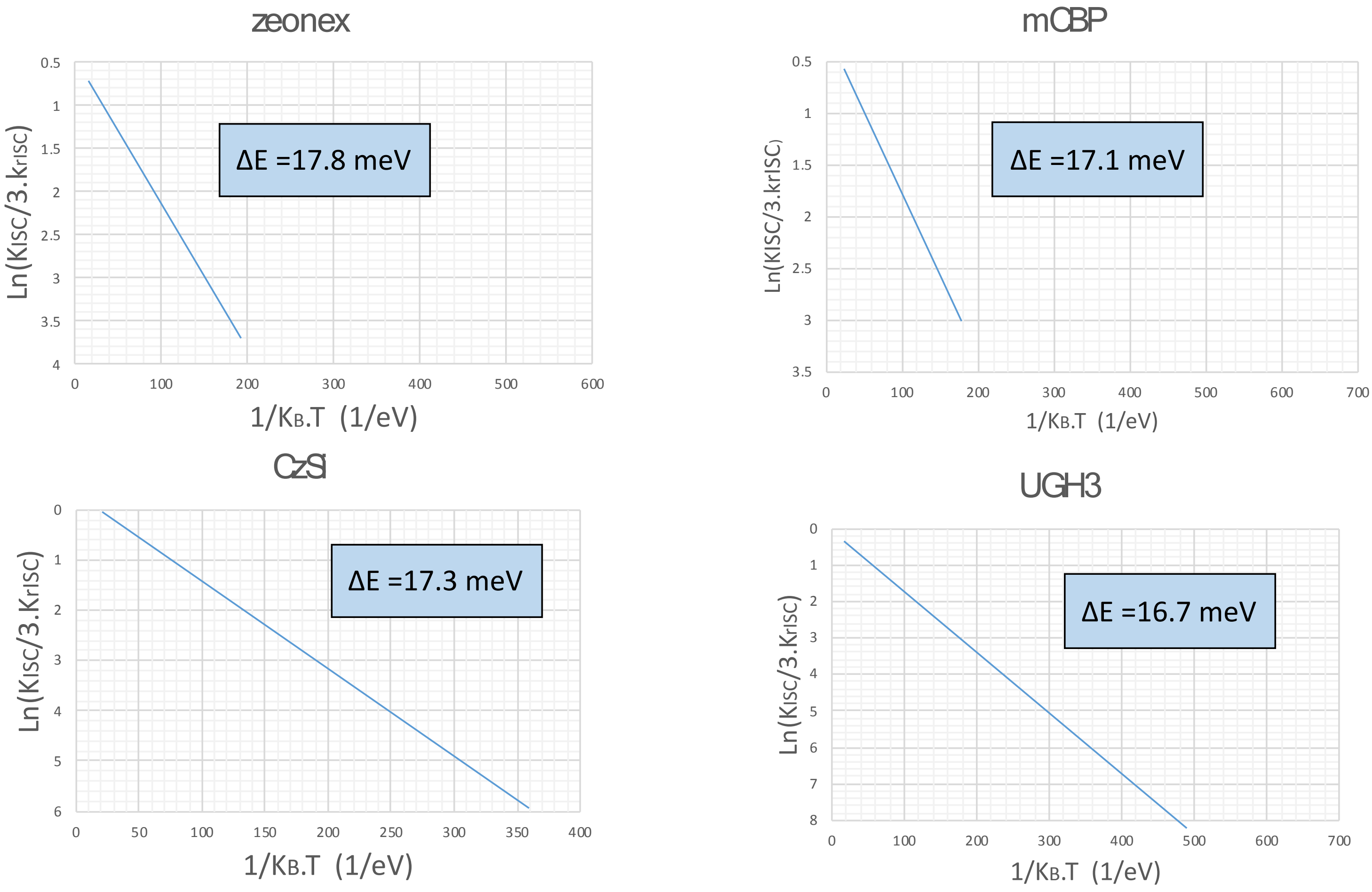

Figure S22. *Plots of* $\frac{k_{rISC}}{k_{ISC}} = \frac{1}{3}exp\left[-\frac{\Delta E_{ST}}{k_B T}\right]$ *to estimate the non-adiabatic energy gap between coupling CT and LE triplet states.*

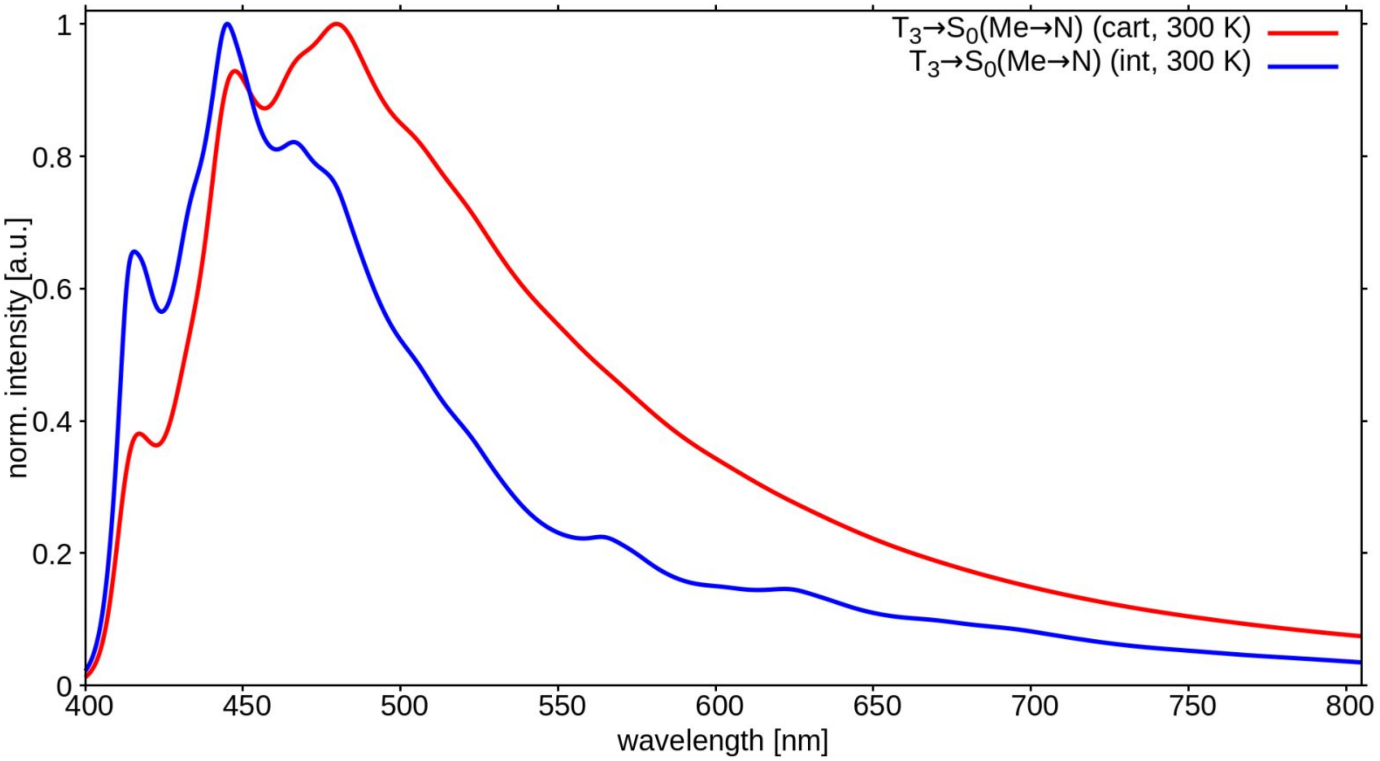

Figure S23 Theoretically calculate phosphorescence spectrum of the T_3 lowest energy local triplet state at 77K. Spectra in cartesian and internal coordinates are shown. The internal mode spectra are the most representative of what should be observed experimentally.

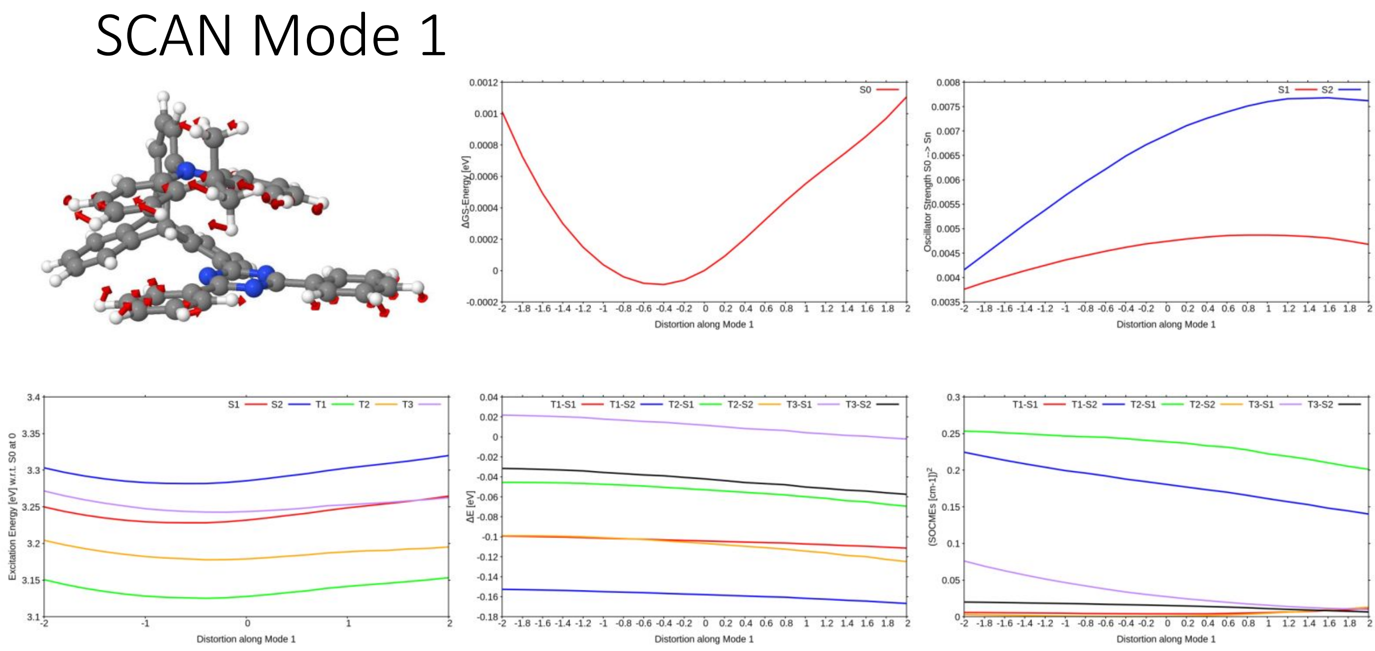

Figure S24 Vibrational mode that intercouples the two lowest energy TpAT-tFFO conformers

SCAN Mode 12

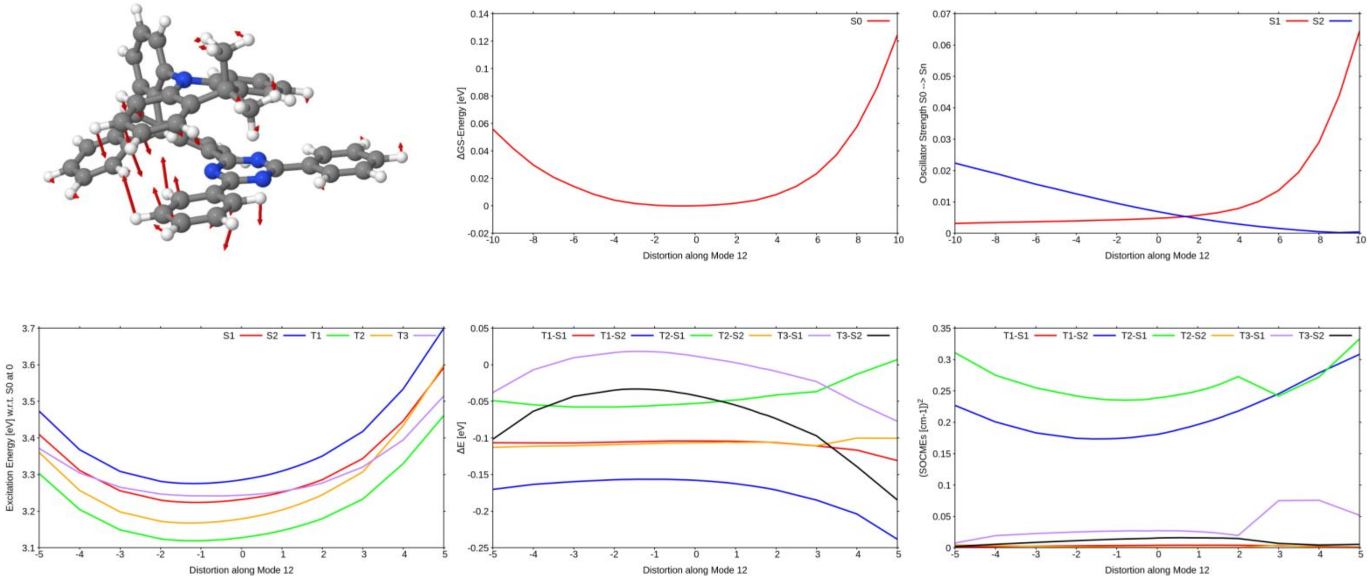

SCAN Mode 9

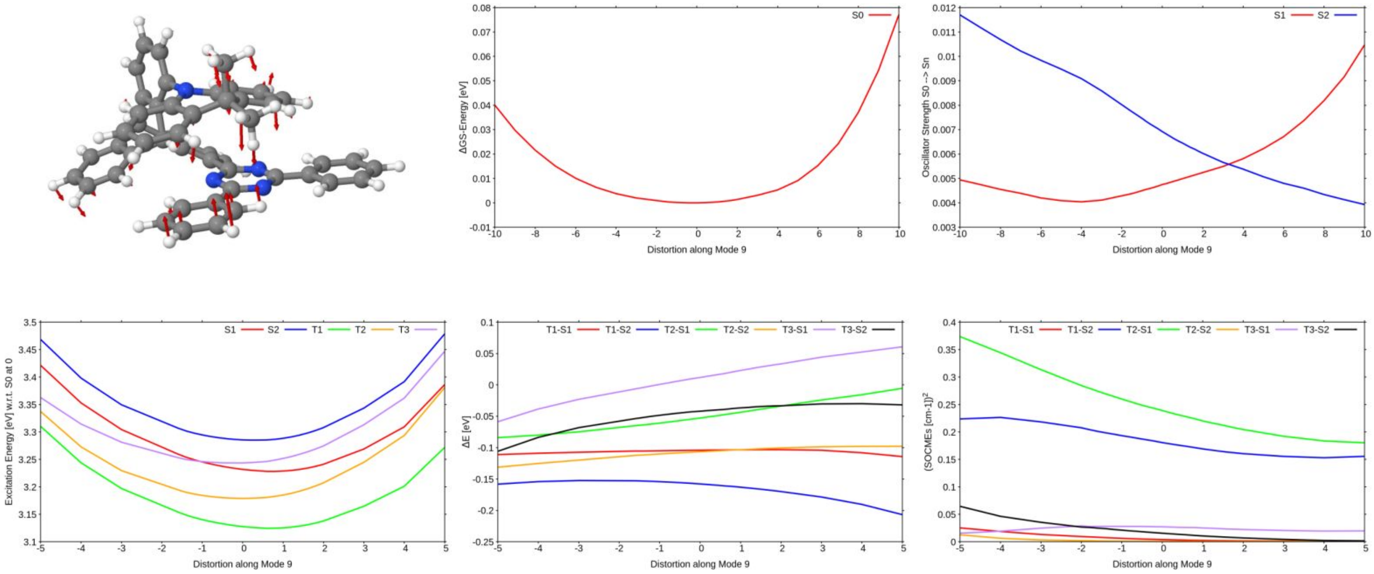

Figure S25 Vibrational modes that have major effect on D A wavefunction overlap and hence singlet triplet energy gap and oscillator strength. TpAT-tFFO conformers

Table S2 Rate constants for the different temperatures of the CzSi matrix time resolved measurements

**CzSi** (10%)			
T [K]	K_F [10^5 s^{-1}]	K_{ISC} [10^6 s^{-1}]	K_{rISC} [10^5 s^{-1}]
300	3.95 ± 0.22	6.94 ± 0.3	14.48 ± 0.05
150	5.42 ± 0.03	5.10 ± 0.10	2.83 ± 0.14
80	5.72 ± 0.51	1.54 ± 0.10	0.44 ± 0.04
50	6.80 ± 0.06	1.00 ± 0.06	0.10 ± 0.01

Table S3 Rate constants for the different temperatures of the UGH-3 matrix time resolved measurements

**UGH-3** (10%)			
T [K]	K_F [10^5 s^{-1}]	K_{ISC} [10^6 s^{-1}]	K_{rISC} [10^5 s^{-1}]
300	5.37 ± ~	14.915 ± 0.24	26.06 ± 0.27
80	3.60 ± 0.28	1.74 ± 0.09	0.54 ± 0.04

Table S4 Rate constants for the different temperatures of the Zeonex matrix time resolved measurements

**Zeonex** (1%)			
T [K]	K_F [10^5 s^{-1}]	K_{ISC} [10^6 s^{-1}]	K_{rISC} [10^5 s^{-1}]
300	5.28 ± 0.15	18.88 ± 0.05	2.09 ± 0.96
150	5.32 ± 0.04	7.47 ± 0.44	3.55 ± 0.03
80	9.49 ± 0.10	2.88 ± 0.16	0.54 ± 0.04
50	4.83 ± 0.43	2.39 ± 0.12	0.19 ± 0.01
